# *L*_*p*_-Adaptation: Simultaneous Design Centering and Robustness Estimation of Electronic and Biological Systems

**DOI:** 10.1038/s41598-017-03556-5

**Published:** 2017-07-27

**Authors:** Josefine Asmus, Christian L. Müller, Ivo F. Sbalzarini

**Affiliations:** 1 Scientific Computing for Systems Biology, Faculty of Computer Science, TU Dresden, 01069 Dresden, Germany; 2 Center for Advancing Electronics Dresden (cfaed), TU Dresden, 01069 Dresden, Germany; 3 MOSAIC Group, Center for Systems Biology Dresden (CSBD), 01307 Dresden, Germany; 4 Flatiron Institute, Simons Foundation, New York, 10010 USA; 5 Max Planck Institute of Molecular Cell Biology and Genetics, 01307 Dresden, Germany

## Abstract

The design of systems or models that work robustly under uncertainty and environmental fluctuations is a key challenge in both engineering and science. This is formalized in the design-centering problem, which is defined as finding a design that fulfills given specifications and has a high probability of still doing so if the system parameters or the specifications fluctuate randomly. Design centering is often accompanied by the problem of quantifying the robustness of a system. Here we present a novel adaptive statistical method to simultaneously address both problems. Our method, *L*_*p*_-Adaptation, is inspired by the evolution of robustness in biological systems and by randomized schemes for convex volume computation. It is able to address both problems in the general, non-convex case and at low computational cost. We describe the concept and the algorithm, test it on known benchmarks, and demonstrate its real-world applicability in electronic and biological systems. In all cases, the present method outperforms the previous state of the art. This enables re-formulating optimization problems in engineering and biology as design centering problems, taking global system robustness into account.

## Introduction

Design centering is a long-standing and central problem in systems engineering and model inference. It is concerned with the determination of design parameters for a system or model that guarantee satisfactory operation within given specifications, and which are robust against random variations. While *design optimization* aims to determine the design that *best* fulfills the specifications, it is the aim of *design centering* to find the design that meets the specifications most *robustly*. Traditionally, this problem has been considered in electronic circuit engineering^1^, where a typical task is to determine the nominal values of electronic components (e.g., resistances, capacitances, etc.) such that the circuit fulfills some specifications and is robust against manufacturing tolerances in the components. Examples of specifications in electronic circuits are frequency response, harmonic distortion, energy consumption, and manufacturing cost. Recently, related ideas have also entered the field of synthetic biology with the aim of robustly designing novel synthetic biological circuits^2, 3^. Any criterion that can be verified for a given design can be used as a specification.

In order to be robust against perturbations, the specifications cannot be too narrowly defined. This implies that there are usually many designs that fulfill the specifications. We refer to this set of satisfactory designs as the *feasible* designs. The size or volume of the set of all feasible designs is an intuitive measure for the robustness with which the specifications can be fulfilled. Robustness is therefore related to the probability that the design still fulfills the same specifications when the design parameters randomly vary, or the specifications fluctuate. Quantifying this robustness requires estimating the size or volume of the set of all feasible designs.

A fundamental requirement for volume estimation and design centering is the existence of a procedure (referred to as “oracle”)^4^ that can check whether a given design fulfills the specifications, or not. In this general setting, design centering and volume estimation are hard problems. Exhaustive enumeration of all feasible designs requires exponentially many design trials in the number of design parameters. Since typical systems or circuits have tens to hundreds of design parameters, testing all possible combinations is prohibitive. It is hence intuitive that many design centering problems are NP-hard^5^, i.e., they cannot be efficiently solved on a deterministic computer. Surprisingly, it is also NP-hard to approximate the volume of a high-dimensional set using a deterministic algorithm^6, 7^, even if the set is convex. Efficient approaches to design centering and volume estimation are therefore always stochastic. However, even though volume estimation and design centering are closely related problems, previous approximate approaches have considered them separately^8–17^.

Here, we jointly consider the problems of design centering and volume estimation in their most general form, only requiring an oracle description of the underlying system. We present a statistical framework that unites the two problems and introduce an efficient randomized algorithm, called *L*_*p*_-Adaptation, for the practical application of this new framework.

The proposed conceptual framework highlights several links and tradeoffs between design centering and volume estimation. It is inspired by the observation that robust designs are a hallmark of biological systems, such as cell signaling networks, blood vasculature networks, and food chains^18^. Biological systems need to be robust against fluctuations, otherwise they would be less likely to survive in a changing environment. It has been observed that the robustness of biological networks is related to the volume of the set of feasible parameters^19, 20^. This is the same definition of robustness used for engineering systems. It appears that nature has found a way of approximating both design centering and volume estimation through self-organization and natural selection. This succession of design alteration and design selection is akin to bio-inspired optimization algorithms, such as evolution strategies^21^ and genetic algorithms^22^, with the important difference that it is not optimization that is the goal, but rather design centering and volume estimation. In our framework, design selection is performed by checking whether the specifications are fulfilled. Feasible designs then undergo random alterations with the specific aim of exploring the space of all feasible designs as broadly and efficiently as possible.

Efficient and broad exploration of feasible designs is the core of the *L*_*p*_-Adaptation algorithm. Following the biological inspiration, the algorithm is based on adaptive stochastic sampling of designs together with a consistent way of converting the explored samples to an estimate of the robustness and the design center. As we show below, *L*_*p*_-Adaptation is computationally efficient, equalling or outperforming the previous state of the art. Most importantly, however, *L*_*p*_-Adaptation is based on the joint consideration of the two problems and therefore relaxes the limiting assumptions made by previous approaches about either the convexity or smoothness of the set of feasible designs, or the correlations between parameters. *L*_*p*_-Adaptation provides a computationally efficient and versatile method for approximately solving general, oracle-based and non-convex design-centering and volume estimation problems.

## Statement of the Problem and Previous Approaches

We start by formalizing the design centering problem and reviewing previous approaches. Without loss of generality, and to simplify the notation, we assume parametric designs. These are designs that are described by a (possibly large) number *n* of numerical parameters. Non-parametric designs can always be reduced to parametric ones by appropriate parametrization or discretization, which is anyways necessary for implementation in a digital computer. We can thus use the words “design” and “parameters” synonymously and consider the design (or parameter) space to be ${{\mathbb{R}}}^{n}$, i.e., the *n*-dimensional vector space of real numbers.

The region or subspace of the parameter space that contains all parameter vectors for which the system meets or exceeds the specifications is called the *feasible region* $A\subset {{\mathbb{R}}}^{n}$. We denote the total volume of the feasible region by *V* = vol(*A*), defined as the integral of the uniform density over *A*. This volume is a natural measure for the total number of feasible designs available and can be used to compare and choose between different designs or competing models^23^. Moreover, the overall shape and orientation of the feasible region contains information about correlations between design parameters, which can be exploited for model reduction and to guide the experimental verification of a design.

Depending on the specific design scenario, different operational definitions of the *design center* **m** ∈ *A* exist, including the *nominal design center*, the *worst-case design center*, and the *process design center* ^8^. For instance, in the example of manufacturing an electronic circuit from components with known manufacturing tolerances, the design center is the nominal parameter vector that maximizes the production yield. Here, we adopt a general statistical definition of the design center^9, 24^. Among all feasible points (parameter vector) **x** ∈ *A* the statistical design center **m** ∈ *A* is the mean of a parametric probability distribution *p*(**x**) of maximal volume which covers the feasible region *A* with a given *hitting probability P*. For convex feasible regions, using the uniform probability distribution over *A* and *P* = 1, the design center coincides with the geometric center of the feasible region.

Previous approaches to design centering can be classified into geometrical and statistical approaches^10^. Geometrical approaches use simple geometric bodies to approximate the feasible region, which is usually assumed to be convex^25^. Examples of geometrical approaches include simplicial approximation^11, 12^, which approximates the boundary of the feasible region by adaptation of a convex polytope. Due to the curse of dimensionality, however, simplicial approximation becomes unpractical in dimensions *n* > 8^26, 27^. Suggested improvements to relax the convexity requirement instead assume differentiability of the specifications^28^, which cannot be guaranteed in black-box problems. Another example of a geometrical approach is the ellipsoidal approximation^13^, which finds the ellipsoid of largest volume that still completely fits into the feasible region. All endpoints of the ellipsoidal axes and the center of the ellipsoid need to be feasible. While the ellipsoidal approximation does not strictly require convexity of the feasible region, its approximation properties strongly depend on it. More recently, ellipsoidal approximation has been accelerated using surrogate models to approximate the oracle function^29^. A third example of a geometrical approach is the polytope method^10^, which also uses a convex polytope to approximate the feasible region, but then finds the design center by either inscribing the largest Hessian ellipsoid or by using a convex programming approach. The latter approach, however, requires an explicit probabilistic model of the variations in the design parameters, which is usually not available in practice.

Statistical approaches approximate the feasible region by Monte Carlo sampling. Since exhaustive sampling is not feasible in high dimensions, the key ingredient of statistical methods is to find an effective sampling proposal, and to concentrate on informative regions. The methods then sample points from this proposal and evaluate the specifications for these points to decide if they are feasible. The ratio of feasible to infeasible points sampled then provides information about the robustness of a design^14^. Constraint adaptation by differential evolution (CADE)^15^ is a classical statistical design-centering method based on differential evolution^30^. It assumes the feasible region to be convex and starts from a population of initial points. To find those points, the specifications (constraints) are relaxed and then tightened successively to the original ones. After the original specifications are met, the mean of all points, which have to be feasible, is used as an approximation of the design center. Another statistical approach is the advanced first-order second moment (AFOSM) method^8^. It samples candidate points from *L*_*p*_-balls in order to estimate the yield (i.e., the ratio of feasible to infeasible points) and approximate the feasible region. Which *L*_*p*_-norm to use is directly related to the assumed statistical distribution of the random perturbations. The proposal *L*_*p*_-balls are adapted to maximize their volume while still being completely contained within the feasible region. This does not allow estimating the total volume of the feasible region. A third example of a statistical method is the center of gravity method^26^. In each iteration the centers of gravity of the feasible samples and of the infeasible samples are computed. The design center is then moved toward the center of the feasible points and away from the center of the infeasible ones. The momentum-based center of gravity method^16^ extends this idea to include information from the past *two* iterations. A more recent statistical approach considered design centering in cases where the variables are correlated, and the perturbations are normally^31^ or non-normally^32^ distributed.

## ***L***_***p***_-Adaptation: An Efficient Method Uniting Approximate Design Centering and Volume Estimation

**Figure 1:**
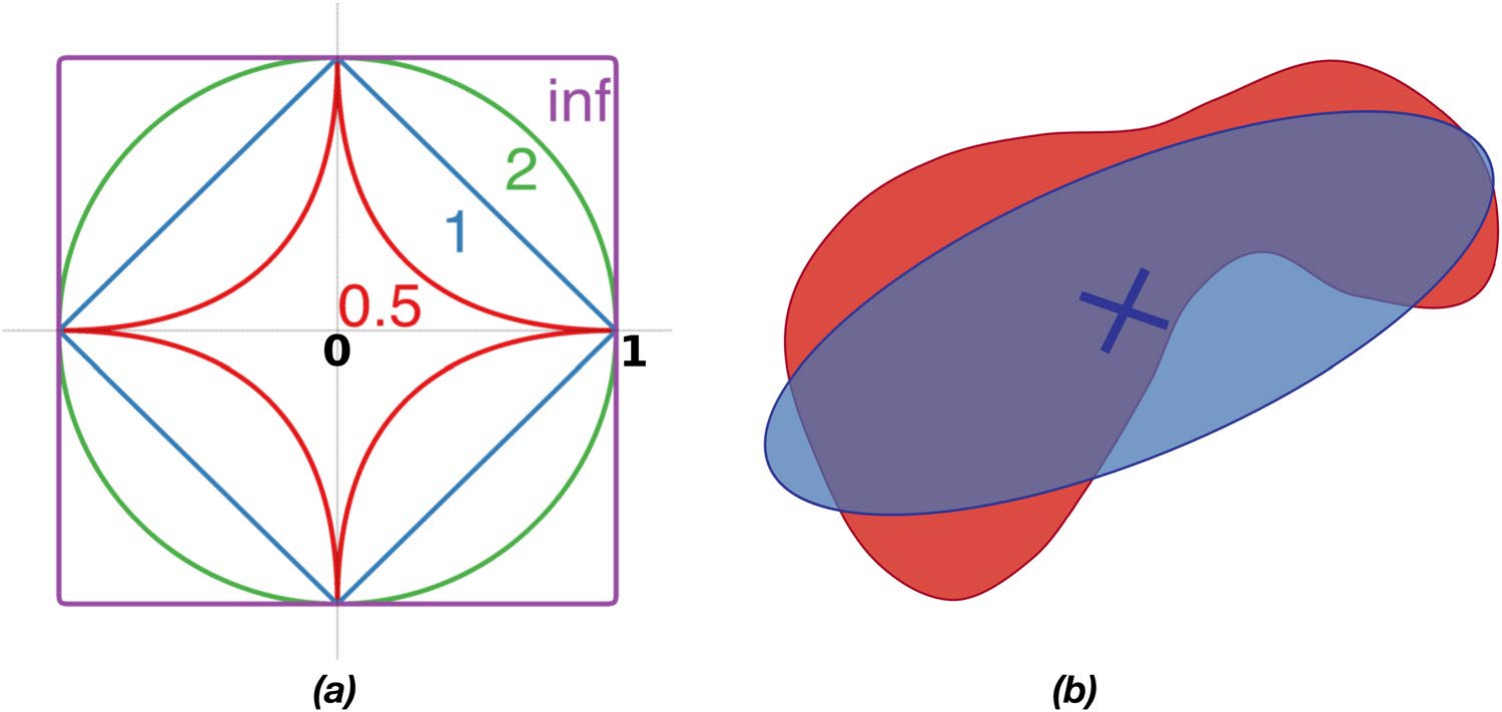
(**a**) Illustrations of some unit *L*_*p*_-balls in two dimensions: *p* → ∞: the *L*_∞_-ball (rectangle), *p* = 2: the *L*_2_-ball (circle), *p* = 1: the *L*_1_-ball (diamond), and *p* = 0.5: the *L*_0.5_-ball (star). (**b**) Samples are randomly drawn from the proposal distribution (blue), which is an affine transformation of an *L*_*p*_-ball. The hitting probability *P* is the probability that the sample lies inside the feasible region (red). For uniform sampling, *P* is given by the overlap area between the proposal and the feasible region.

We propose a novel statistical method, termed *L*_*p*_-Adaptation, that unites approximate design centering and volume estimation. The method iteratively samples the parameter space using the uniform density over *L*_*p*_-balls as proposal distribution *p*(**x**). Some examples of *L*_*p*_-balls in two dimensions are illustrated in Fig. 1a. In contrast to classical Markov Chain Monte Carlo methods, our algorithm dynamically adapts position, orientation, and aspect ratio of the *L*_*p*_-balls based on the sampling history. This is inspired by adaptation concepts introduced in bio-inspired optimization^9, 33^ and leads to better sampling efficiency and approximation quality, especially in feasible regions of high aspect ratio. While non-convexity will deteriorate sampling efficiency, our approach is not limited to convex feasible regions, since it can dynamically adapt to different areas of the feasible region.

The dynamic, affine adaptation of the *L*_*p*_-ball is based on the concept of Gaussian Adaptation (GaA)^9^, which continuously adapts the mean and the covariance matrix. The covariance matrix describes correlations and scaling between the parameters of a Gaussian proposal based on previous sampling success. GaA is a classical optimization heuristic that is linked to maximum-entropy estimation^34^ and provides a unifying algorithmic framework for optimization and sampling^35^. Based on this analogy, GaA has previously been extended to design centering^36^. The use of a Gaussian proposal, however, becomes unfavorable for feasible regions of non-elliptic shape.

Combining the adaptation concept of GaA with the use of *L*_*p*_-balls^8^ as non-Gaussian proposals, we show here that efficient design centering and robust volume approximation become possible in the same framework. *L*_*p*_-Adaptation draws samples uniformly from an *L*_*p*_-ball and iteratively adapts position, orientation, and aspect ratio of the *L*_*p*_-ball. This is done by adapting the mean and covariance matrix of an affine mapping applied to the balls prior to sampling. An important feature of the sampling and adaptation process is its ability to control the *target hitting probability P*, i.e., the probability of hitting the feasible region *A* with a sample. This allows *L*_*p*_-Adaptation to provide instantaneous design center and volume estimates. The design center is approximated by the mean of the current *L*_*p*_-ball, and the volume estimate is of the form vol(*A*) ≈ *P* ⋅ vol$({L}_{p}^{n})$, where vol$({L}_{p}^{n})$ is the volume of the current *n*-dimensional *L*_*p*_-ball. For improved sampling and adaptation efficiency, at each iteration *L*_*p*_-Adaptation uses an adaptive multi-sample strategy^37^ that is considered state-of-the-art in bio-inspired optimization^33^. The details of the algorithm are given in Supplementary Note 1.

**Figure 2:**
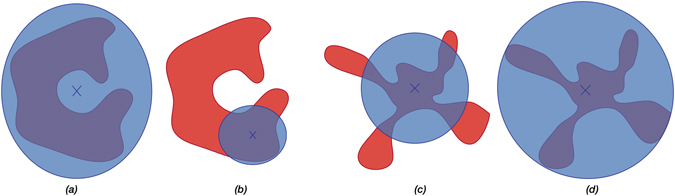
Illustration of the effect of different hitting probabilities on a feasible region. (**a**) Low hitting probabilities may lead to infeasible design centers. (**b**) Increasing the hitting probability (e.g. shrinking the proposal) leads to a feasible design center. (**c**) For volume estimation, high hitting probabilities may under-estimate the volume. (**d**) Decreasing the hitting probability leads to better volume approximation.

In addition to adapting the sampling proposal, we also adapt the target hitting probability *P* of the sampler to the task at hand. This is illustrated in Fig. 1b. The hitting probability must be neither too low, nor too high. Low hitting probabilities lead to low sampling efficiencies. High hitting probabilities lead to a slower adaptation to the feasible region, which may prevent exploring remote parts of the feasible region. This trade-off is illustrated in Fig. 2. For a Gaussian proposal and a convex feasible region, a hitting probability of 1/e is information-theoretically optimal^9^, where e is the inverse of the Euler number. When sampling uniformly from *L*_*p*_-balls over non-convex regions, however, no such result is available. We therefore dynamically adapt the hitting probability depending on the task at hand, starting from 1/e as an initial value. For design centering, the hitting probability in non-convex regions cannot be too low, as this could lead to an infeasible design center (Fig. 2a). We therefore successively increase the hitting probability in order to drive the process toward a random feasible design center (Fig. 2b). For volume approximation the hitting probability must not be too high, otherwise we would miss some parts of the feasible region (Fig. 2c). In this case, the hitting probability is successively lowered until the volume estimate no longer changes (Fig. 2d), leading to a better volume approximation. This strategy is similar to state-of-the-art multi-phase Monte Carlo methods for approximate convex volume estimation^38, 39^. Details about the adaptation and volume computation procedure are given in Supplementary Note 1.

## Evaluation and Application

We demonstrate the properties and application of *L*_*p*_-Adaptation in six examples. The first four are benchmark cases where the correct answers are known or comparisons from the literature are available. For this, we consider two simple 2D test cases from the literature, followed by basic *L*_*p*_-balls in up to 20 dimensions. The last two examples are real-world applications, one from electronics and one from systems biology, where the true answer is unknown, but comparisons from the literature are available.

### Illustration in two dimensions

**Figure 3:**
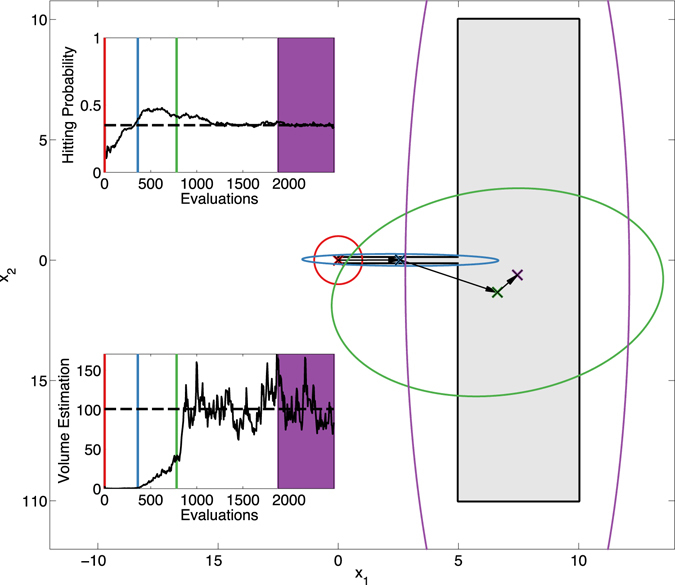
Illustration of *L*_*p*_-Adaptation using the test case of Storn^15^. The feasible region (shaded gray) is non-convex and deceiving, consisting of a large rectangle with a thin arm attached to it. The algorithm is started from the tip of the arm, which is an unfavorable starting point (red). Even though it initially cannot “see” the large rectangle, the proposal distribution adapts to the shape of the arm (blue) within 355 evaluations and discovers the large rectangle after 775 evaluations (green). The evolution of the design center is shown by crosses, the *L*_2_ proposal is shown as ellipses. Colors correspond to the evaluations as indicated. The two inset plots show the evolution of the hitting probability and the volume estimate versus the number of function evaluations. The final design center and volume (purple) are obtained by averaging over the last 600 evaluations.

Figure 3 shows the behavior of *L*_*p*_-Adaptation in a classic 2D test case from the literature^15^. The feasible region is shaded in gray. It consists of a large rectangle (*x*_2_ ∈ [−10, 10] and *x*_1_ ∈ [5, 10]) with a thin attached arm (*x*_2_ ∈ [−0.1, 0.1] and *x*_1_ ∈ [0, 5]). The region is not convex, and the thin arm is deceiving for an algorithm. We start *L*_*p*_-Adaptation from a feasible point at the very tip of this arm, at (0, 0) (red cross in Fig. 3). This is a particularly unfavorable starting point for both design centering and volume estimation of this feasible region. The initial proposal distribution (red ellipse) is isotropic with a radius of 1, which does not allow the algorithm to “see” the large rectangle initially. After 355 function evaluations, however, the proposal distribution has adapted to the shape of the thin arm (blue ellipse) and points in the large rectangle are first found. This progressively moves the center of the proposal toward the large rectangle, which is reached after 775 evaluations (green ellipse). The algorithm is run until 2500 evaluations, when it has reached a stationary state (stationary random process). This means that the statistics of the proposal distribution, such as hitting probability and mean volume, no longer change on average, although the samples still stochastically fluctuate. Averaging over the last 600 evaluations (purple areas in the inset figures) provides the final estimates of the design center and volume of the feasible region. Since the process converges in distribution, i.e., the random process becomes stationary, averaging is meaningful. The final volume estimate is 100.8 (true value: 101.0) and the design center is (7.45, −0.62). This compares well with the exact center of the large rectangle, which is (7.5, 0.0). Any point with *x*_1_ = 7.5 and *x*_2_ ∈ [−7.5, 7.5] has maximum distance from any border of the feasible region.

Before the proposal distribution has adapted to the shape of the thin arm, the effective, empirical hitting probability is lower than the target hitting probability (dashed line in the inset figure). Thereafter, the hitting probability increases until the mean of the proposal distribution has moved into the large rectangle. After around 1200 evaluations the target hitting probability is reached and maintained. The lower inset plot shows how the algorithm “discovers” the large part of the feasible region. Around 775 evaluations (green line), the estimated volume sharply increases as the large rectangle becomes visible, and it finally fluctuates around the true value of 101.0 (dashed line).

**Figure 4:**
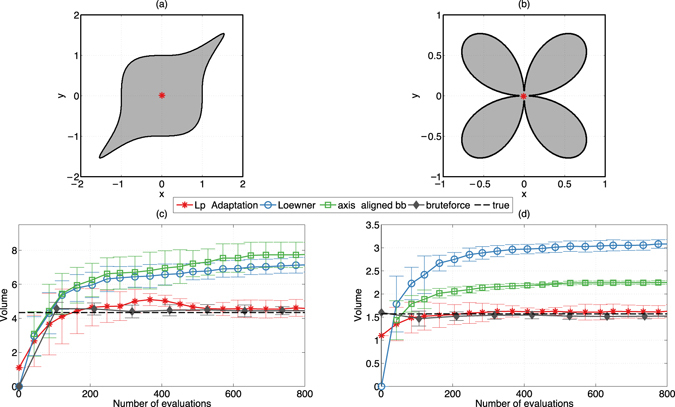
Two 2D test cases from the literature: (**a**) the “Handle”^40^, given by the parametric equation *x* ^6^ + *y* ^6^ − 1.925*x* ^3^ *y* ^3^ ≤ 1, and (**b**) the “Folium”^41^ parameterized by −(*x* ^2^ + *y* ^2^)^3^ + 4*x* ^2^ *y* ^2^ ≥ 0. Both feasible regions (shaded gray) are non-convex. The red dots are the averaged design centers (of ten individual runs). (**c**,**d**) Average and standard deviation (over ten independent runs) of the volume estimates obtained with *L*_*p*_-Adaptation (red stars) and brute-force exhaustive sampling (gray diamonds). Upper bounds are obtained from the Loewner ellipsoid (blue circles) and axes-aligned bounding box (green square) of the samples generated by *L*_*p*_-Adaptation. The true volume is indicated by the dashed black line.

Figure 4 shows two other classic 2D test cases from the literature: the “Handle”^40^ (Fig. 4a) and the “Folium”^41^ (Fig. 4b). Again, the feasible regions are shaded in gray. The volume approximations obtained by different methods are shown in the panels below. *L*_*p*_-Adaptation (red stars) uses an ellipsoidal proposal distribution (*p* = 2) and starts from ten random feasible points. Results for other *L*_*p*_-balls and for higher numbers of function evaluations are shown in Supplementary Note 2. The results are compared with those from uniform sampling (“bruteforce”, gray diamonds) and with the upper bounds provided by the Loewner ellipsoid^42^ (blue circles) and the axes-aligned bounding box (green squares) of all feasible samples. The true volume is indicated by the dashed black line. The Loewner ellipsoid^42^ of a set of points is the unique minimal-volume ellipsoid that contains the set of points. Looking at its evolution over time shows how well the feasible region is explored. As long as the volume of the Loewner ellipsoid is increasing, new parts of the feasible region are still being found. The large variances of the Loewner ellipsoids and the axes-aligned bounding boxes for the “Handle” indicate that the individual runs differ in how well they explore the outermost arms of the handle.

Brute-force sampling provides the most accurate result, but is exponentially inefficient with dimension. After around 600 evaluations for the “Handle” and 150 evaluations for the “Folium”, the correct volume is in the error bars of the results from *L*_*p*_-Adaptation. The final centers of the estimated volumes are shown by red dots in the upper panel of Fig. 4. In both cases they are at the geometric center of the body. However, the geometric center of the estimated volume need not be a robust design center, as evident for the “Folium”. Taken together, these results suggest that *L*_*p*_-Adaptation provides volume estimates that are as good as exhaustive sampling already in two dimensions. In addition, dynamic computation and monitoring of Loewner ellipsoids and axis-aligned bounding boxes of the samples (i) give practical upper bounds on the approximate volume, (ii) allow the definition of additional convergence criteria, and (iii) provide additional global geometric insight about the feasible region. For instance, the Lowner ellipsoid gives a tighter bound on the “Handle” than the axis-aligned bounding box due to better approximation of an ellipsoid to the “Handle”, while for the “Folium” the axis-aligned box is preferred.

### Benchmark on Synthetic *L*_*p*_-Balls

In order to test the algorithm in higher dimensions, we consider *L*_*p*_-balls as feasible regions. Unlike the fixed-dimensional test cases from the literature, these can be scaled to arbitrary dimension with the true result still known in all cases. We use *L*_*p*_-Adaptation to estimate the volumes of these *L*_*p*_-balls, which is particularly interesting when the true *p* and the proposal *p* do not match. We also test GaA^36^ on all test bodies and the state-of-the-art convex volume estimation algorithm of Cousins and Vempala^17^ for *p* = 1, 2, ∞.

**Figure 5:**
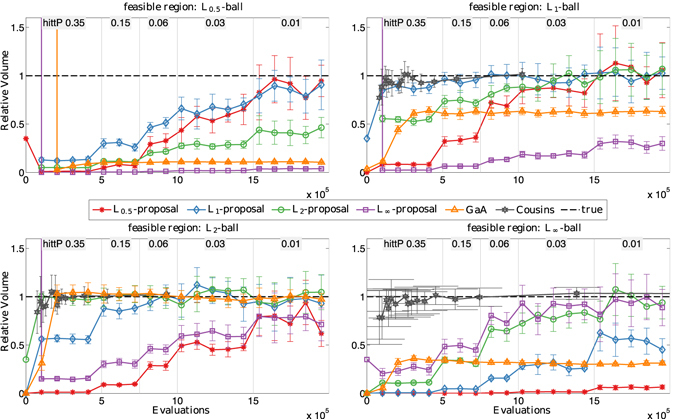
Average and standard deviation (over ten independent runs) of the normalized volume (estimated volume/true volume) of anisotropic 20-dimensional *L*_*p*_-balls (*p* = 0.5, 1, 2, ∞) approximated with proposal distributions of different *p*. The results are shown for decreasing target hitting probability, starting from the default initialization 0.35, as indicated at the top of each plot. The dashed line shows the true volume. For comparison, we also show the results obtained with Gaussian Adaptation^36^ and Cousins’ convex volume estimator^17^.

Figure 5 shows the estimated volumes for different feasible regions (p-norms 0.5, 1, 2, and ∞) when using different proposals (proposal p-norms 0.5 (red), 1 (blue), 2 (green), and ∞ (purple)) in 20 dimensions. The true volume is indicated by the dashed black line. All feasible regions are stretched along (*n* − 1) axes such that the longest axis is $\sqrt{1000}$ times longer than the shortest one, and the lengths of the axes are logarithmically spaced. Since *L*_*p*_-Adaptation starts with an isotropic proposal, this anisotropy of the feasible regions first needs to be “learned” by the adaptation mechanism. The figure shows the volume estimates versus the number of function evaluations. We initialize with the standard target hitting probability 0.35 in order to learn the shape of the feasible region. As indicated at the top of the plots, we then successively lower the target hitting probability to 0.15, 0.06, 0.03, and 0.01 in order to refine the volume estimate. In general, the results are best when the proposal *p* matches the shape of the feasible region. In practical applications, however, the true shape of the feasible regions is unknown. It is therefore interesting to observe that the *L*_1_- or *L*_2_-ball is a good choice in all cases.

Compared to GaA with fixed hitting probability 0.01, *L*_*p*_-Adaptation works better in all cases. GaA consistently underestimates the volume except when the feasible region is an *L*_2_-ball, which matches the shape of the Gaussian proposal. The convex volume estimation algorithm of Cousins and Vempala^17^ equals or outperforms *L*_*p*_-Adaptation for convex *L*_*p*_ balls. This is expected because the algorithm is specialized in convex bodies and has direct access to the underlying geometric description of the body (a polytope or an ellipsoid) whereas *L*_*p*_-Adaptation requires no prior knowledge. Remarkably, *L*_*p*_-Adaptation shows similar convergence for *L*_1_-balls and *L*_2_-balls when using the same bodies as proposals. We conclude that *L*_*p*_-Adaptation performs better than Gaussian Adaptation and reaches the same accuracy as specialized algorithms for convex bodies, albeit without requiring convexity or prior knowledge about the shape of the feasible region.

### Case Studies on Real-World Problems

In addition to tests with known ground truth, we demonstrate the application of *L*_*p*_-Adaptation to representative real-world cases. The true solution is not known for these cases, but they have previously been considered in the literature. This allows us to compare our results. We first consider an application from electronics, where the goal is robust design centering, followed by an example from systems biology, where the goal is robust parameter inference and model selection by robustness quantification. We show how *L*_*p*_-Adaptation can be used for both questions.

#### Switched Capacitor Filter

**Figure 6:**
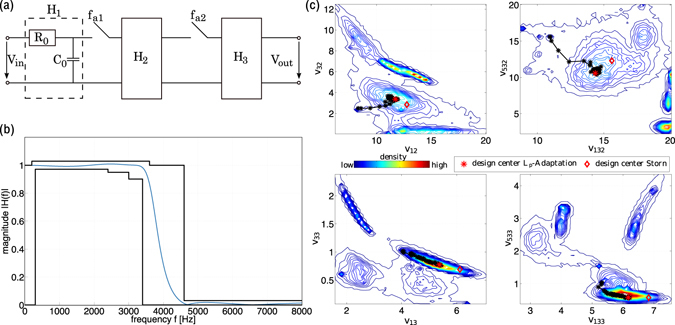
(**a**) Circuit diagram of the Switched Capacitor (SC) filter. (**b**) Specifications for the transfer function of the filter (black lines) and one example satisfying the constraints (blue line). (**c**) Pairwise density contour plots of the feasible points obtained by ten runs of *L*_*p*_-Adaptation with different starting points in the nine-dimensional design space. The black trajectory show the evolution of the mean for the run that yielded the largest volume approximation. The red star is the final design center obtained by that run. The design center found by Storn^15^ in the same part of the feasible region is indicated by the red diamond.

Switched Capacitor (SC) filters are a modern replacement for Resistor Capacitor (RC) filters. They are well suited for integration on silicon chips, due to reduced sensitivity of their transfer function to manufacturing inaccuracies. While the basic design processes of SC and RC filters are similar, a key challenge in SC filter design are parasitic capacitances. Here, we consider a design scenario for an SC-based pulse code modulation (PCM) low-pass filter with parasitic capacitances, as introduced as a test case by Storn^15^. The circuit diagram of this example is shown in Fig. 6a. The transfer function of this SC-PCM filter is:

1 $$H(f)=\frac{{V}_{{\rm{out}}}}{{V}_{{\rm{in}}}}={H}_{1}\cdot {H}_{2}\cdot {H}_{3},$$

where *f* is the frequency of the signal, *H*_1_ the transfer function of the analog RC filter at the input and *H*_2_, *H*_3_ of the two SC blocks as shown in Fig. 6a. The particular expressions for *H*_1_, *H*_2_, and *H*_3_ with parasitic capacitances are given in Supplementary Note 3. According to these expressions, the filter has nine design parameters: {*v*_1_, *v*_12_, *v*_32_, *v*_132_, *v*_532_, *v*_13_, *v*_33_, *v*_133_, *v*_533_}, defining a 9-dimensional design space. The feasible region consists of all points in that space for which *|H*(*f*)| lies within the specifications given by the solid black lines in Fig. 6b. They define the gain, ringing, and sharpness characteristics of the filter. The mathematical expressions for the specification are given in Supplementary Note 3.

We start *L*_*p*_-Adaptation from ten different initial feasible points found by parameter optimization using the Gaussian adaptation (GaA) algorithm^9, 34^. For this initial optimization, we allow *v*_1_ to vary within the interval [e^−9^, e^3^] and all other parameters in [e^−3^, e^3^]. The objective function is the sum of squared deviations between the realized |*H*(*f* )| and the prescribed specification boundaries of the transfer function across the frequency range *f*. The optimization starts from ten different points that are sampled uniformly in the natural logarithm of the entire parameter domain.

Figure 6c shows four selected pairwise density contour plots of the feasible points obtained by the ten runs of *L*_*p*_-Adaptation. A high density of feasible points is shown in red, low density in blue. The lines are curves of constant density. The black trajectory shows the evolution of the mean of the proposal distribution for the run that yielded the largest volume approximation. Individual iterations of the algorithm are shown by stars. The red star is the final design center found by this run. Because the feasible region in this example is not convex, different runs find different design centers in different parts of the feasible region. The design center found by Storn^15^ in the same part of the feasible region is indicated by the red diamond. We assess the robustness of the design centers found by *L*_*p*_-Adaptation by comparing with the results reported by Storn^15^. Robustness is measured by the size (radius) of a hyper-cube or hyper-ellipsoid that contains a given fraction of feasible points, see Supplementary Figure 5. In all cases, the design centers found by *L*_*p*_-Adaptation are more robust than the previous results.

#### Biological Signaling Network

As a second real-world example, we consider a network model from systems biology: the bacterial two-component system (TCS). The TCS signaling network allows bacteria to sense and respond to environmental changes. The TCS is an evolutionarily conserved stimulus-response mechanism found in all bacterial species, and to a lesser extent also in archaea and eukaryotes such as plants, molds, and yeast.

**Figure 7:**
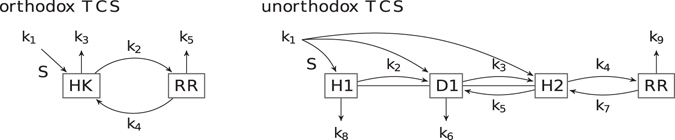
Two different bacterial two-component systems (TCS). Reactions involving phosphate groups are represented by arrows with the respective reaction rates indicated. Left: the “orthodox system”, in which the histidine kinase (HK) auto-phosphorylates upon sensing a stimulus S and passes the phosphate group on to the response regulator (RR) protein. The orthodox system has two phosphate binding domains. Right: the “unorthodox system”, in which the HK has three binding domains: H1, D1, and H2. Together with the binding domain on the RR, this system has a total of four phosphate binding domains.

Stimulus sensing in the TCS relies on a sensor histidine kinase (HK), which auto-phosphorylates and, upon stimulus, passes the phosphate group on to a response regulator (RR) protein. As a response to the stimulus, the phosphorylated RR regulates the transcription of target genes^43^. TCS occur in nature in different flavors, all sharing the same working principle. Here, we compare two TCS differing in the number of phosphate binding domains on the HK. This includes the most commonly found TCS with two binding domains and an alternative form with four stimulus-binding domains. Following the terminology of Barnes *et al*.^2^, we call them the “orthodox system” and the “unorthodox system”, respectively, as depicted in Fig. 7. The ordinary differential equation models describing the dynamics of both systems are given in Supplementary Note 4. The test case compares both systems’ ability to robustly achieve different input-output characteristics by tuning their parameters^2^.

**Figure 8:**
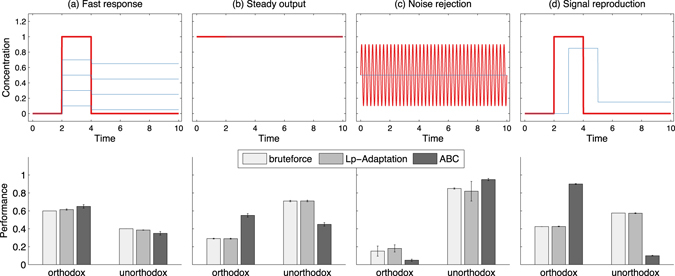
Design centering for four different desired input-output behaviors of the two TCS. Top row: Chemical concentrations as a function of time for the input signal S (red line) and the output signal RR (blue line). For each case, one or several example outputs are shown that fulfill the specifications. Bottom row: The corresponding performances of the orthodox and unorthodox TCS as estimated by three different methods: brute-force sampling, *L*_*p*_-Adaptation, and approximate Bayesian computation based on sequential Monte Carlo (ABC)^2^. Performance is quantified by the posterior probability for each model (ABC) or by the normalized volume (*L*_*p*_-Adaptation and brute-force)^23^. The error bars are computed as described in the main text. (**a**) The “fast response” case requires the network output to react to sudden changes in the input with no more than 0.1 time units delay. (**b**) The “steady output” case requires the output to remain constant at the level of the input at most 2 time units after the input started. (**c**) The “noise rejection” case requires the output to remain constant at the mean of a rapidly fluctuating input. (**d**) The “signal reproduction” case requires the output to reproduce the shape of the input signal with no more than 20% magnitude error and no more than 1 time unit delay.

Figure 8 shows four different desired input-output behaviors, as detailed in Supplementary Note 4 along with the respective design specifications. In the lower panels, the corresponding performances of the orthodox and the unorthodox systems are compared. Performance is quantified by how well a system achieves the task compared to the other system, as computed with three different approaches: brute-force sampling, *L*_*p*_-Adaptation, and approximate Bayesian computation based on sequential Monte Carlo (ABC)^2^. ABC compares the posterior probabilities of the two systems, i.e., the fraction of accepted samples for the given system compared to the total number of accepted samples for both systems. *L*_*p*_-Adaptation and brute-force sampling compare the normalized volumes of the systems, defined as the fraction of the proposal volume for the given system compared to the total volume for both systems. In order to compare models of different dimensionality, volumes are normalized^23^ as $\sqrt[n]{V}$. The error bars for the brute-force sampling are obtained by bootstrapping ten samples of size 5 ⋅ 10^6^ for the orthodox and of size 9 ⋅ 10^6^ for the unorthodox system. The error bars for *L*_*p*_-Adaptation show the standard deviation across ten runs, each with a sample size of 5 ⋅ 10^4^ for the orthodox and 9 ⋅ 10^4^ for the unorthodox system. The error bars for ABC show the variability in the marginal model posteriors over three runs^2^.

The “fast response” case requires the network to rapidly react to abrupt changes in the input stimulus S (Fig. 8(a)). Here, all three approaches reach the same conclusion: both systems are able to achieve the desired input-output response, but the orthodox system outperforms the unorthodox system. Faithfully reproducing a constant input stimulus is the goal of the “steady output” case (Fig. 8(b)). Here, *L*_*p*_-Adaptation agrees with brute-force sampling that the unorthodox system can more robustly produce this behavior, whereas ABC prefers the orthodox system. The “noise rejection” case aims to design a network that reproduces a constant signal while rejecting high-frequency fluctuations about it (Fig. 8(c)). This is much more robustly realized in the unorthodox system, as agreed by all three methods. Finally, the “signal reproduction” case is to design a TCS network where the output signal reproduces the shape of the input stimulus within specified tolerances (Fig. 8(d)). Again, *L*_*p*_-Adaptation agrees with brute-force sampling that the unorthodox system better achieves this behavior, whereas ABC considers the orthodox system better.

**Figure 9:**
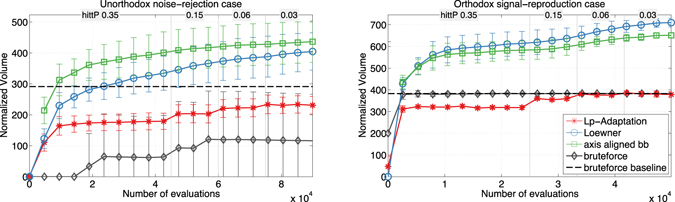
Averages and standard deviations of the normalized volume ($\sqrt[n]{V}$)^23^ estimation of the feasible region over ten independent runs of *L*_*p*_-Adaptation. We show the noise-rejection case for the unorthodox TCS (left column) and the signal-reproduction case for the orthodox TCS (right column) using an *L*_2_-ball proposal. The results for the *L*_1_ proposal are visually indistinguishable and hence omitted. For comparison, the ground-truth baseline obtained by exhaustive brute-force sampling is shown as a dashed black line. Brute-force sampling using the same number of function evaluations as *L*_*p*_-Adaptation is shown in gray. The upper bounds obtained from Loewner ellipsoids and axes-aligned bounding boxes of the *L*_*p*_-Adaptation samples are shown in blue and green, respectively. Their large variance indicates that the feasible region is non-convex or disconnected.

*L*_*p*_-Adaptation can be used for model selection, since the volume of the feasible region is a measure for the robustness of the system. Our results agree with the exhaustive brute-force approach, but only require a fraction of the samples. Figure 9 shows the evolution of the estimated normalized volumes versus the number of function evaluations for the unorthodox model in the noise-rejection case (left) and for the orthodox model in the signal-reproduction case (right). Again, the volumes of the axes-aligned bounding box and the Loewner ellipsoid of all feasible samples are shown as upper bounds. An *L*_2_-ball is used as proposal distribution. The hitting probability is dynamically reduced according to the schedule shown on top of each plot. The dashed black line shows the baseline result obtained by exhaustively brute-force sampling the orthodox system 7.5 ⋅ 10^6^ times and the unorthodox system 1.5 ⋅ 10^7^ times. The results of all individual runs and all eight cases are shown in Suppl. Fig. 6. *L*_*p*_-Adaptation converges toward the baseline in all cases. In the noise-rejection cases, however, the large variances of the Loewner ellipsoids and axes-aligned bounding-boxes suggest that the feasible region is highly non-convex or disconnected. The different runs cover different parts of the non-convex feasible region and some of them do not converge to the baseline. This is manifested in the larger error bars in the left panel of Fig. 9, and clearly visible in the two families of curves in Suppl. Fig. 6.

Nevertheless *L*_*p*_-Adaptation performs much better than brute-force sampling using the same number of function evaluations (gray line) and is able to sample the feasible region much more efficiently. Cases where brute-force sampling reaches the baseline faster are indicative of a feasible region that fills almost the entire space, as confirmed by the large normalized volumes in these cases, i.e. in the fast-response case and in the signal-reproduction case. When the feasible region is significantly smaller than the whole space, *L*_*p*_-Adaptation performs better and more reliably than brute-force sampling (steady-output and noise-rejection cases). This suggests that *L*_*p*_-Adaptation is particularly useful in cases where the feasible region is small compared to the entire search space.

Supplementary Figures 7–10 shows the marginal densities and the joint pairwise distributions of the feasible region for the noise-rejection case of the unorthodox system and for the signal reproduction case of the orthodox system. These are the same cases for which such results have been published before, allowing direct comparison^2^.

Taken together, these results show that *L*_*p*_-Adaptation produces results that agree with the brute-force baseline, albeit at a much lower computational cost. This is true for both design centering and volume estimation.

## Discussion

We have presented *L*_*p*_-Adaptation, a statistical method that unites approximate design centering and volume estimation into a single computationally efficient framework. The method is based on using *L*_*p*_-balls as proposals for sampling, which are dynamically adapted based on the previous samples in order to efficiently explore the feasible region. This feasible region is defined by specifications that the design is required to fulfill. The design center is robust against fluctuations or perturbations in the design parameters and the specifications, with robustness quantified by the volume of the feasible region. The volume of the feasible region is a proxy for the number of feasible designs that exist, and hence provides an intuitive robustness measure. Both design centering and volume estimation are hard computational problems, which have so far been considered separately. To our knowledge, *L*_*p*_-Adaptation is the first algorithm to provide approximate solutions to both problems simultaneously and hence unite them under a single framework.

In addition to providing a common framework for both problems, *L*_*p*_-Adaptation is also computationally efficient. In most cases tested here, *L*_*p*_-Adaptation produced approximations close to ground truth or comparable to exhaustive sampling, albeit at a fraction of the computational cost. In the real-world examples where the feasible region was small compared to the whole design space, orders of magnitude fewer design trials were needed by *L*_*p*_-Adaptation than by exhaustive sampling. In many cases, *L*_*p*_-Adaptation also produced better-quality results than previous approaches. In the real-world example of the switched capacitor filter, for example, the design center that was found had better robustness than the ones previously used as a benchmark, and in all four biological network cases, *L*_*p*_-Adaptation selected the same system as brute-force sampling, whereas previously published results deviated in two cases.

We therefore expect *L*_*p*_-Adaptation to be of practical use, also because it is effectively parameter-free. All algorithm parameters have default settings that only need to be changed in exceptional cases. All results presented here were obtained using the default settings. We also recommend to use *L*_1_ or *L*_2_-balls as default proposal if no prior knowledge is available, since they have shown robust performance. The only requirement for using *L*_*p*_-Adaptation is that a feasible starting point is known. This also makes *L*_*p*_ adaptation an ideal candidate for being used as a base sampler in other parameter exploration methods, which approximate the feasible region by concatenations of ellipsoids^44^ or by approximate posterior distributions through the ABC methodology^2, 45^.

A critical feature of *L*_*p*_-Adaptation in its present form is the sequence of hitting probabilities that is used to steer the process between finding a robust design center and estimating the feasible volume. Successively decreasing the hitting probability allows more accurate volume estimation, yet renders an infeasible design center increasingly likely. In addition, estimating the volume of a high-dimensional (*>*50D) feasible region whose shape does not agree with the used *L*_*p*_-ball can require a very low hitting probability, which may lead to high computational costs. In practice, we thus recommend doing multiple *L*_*p*_-Adaptation runs from different starting points found by initial optimization or brute-force sampling, and to check the quality of the resulting design center and volume estimates. Furthermore, one can also extend the idea of learning position, scale, and orientation of the *L*_*p*_-balls to other convex or quasi-convex bodies, such as closed convex polytopes, that better capture the distribution of the feasible points derived from *L*_*p*_-Adaptation. The only requirement for these alternative (quasi-)convex bodies is the ability to efficiently sample uniform points from them.

The quality of the approximate solutions obtained from *L*_*p*_-Adaptation depends on the unknown shape of the feasible region, the dimensionality of the problem, and the starting point. While the tests presented here are encouraging, we expect *L*_*p*_-Adaptation to underestimate the volume if the feasible region cannot be covered with reasonable hitting probability. An example would be a star-shaped region in high dimensions with heavy arms. In addition, disconnected or thinly connected regions that are well separated may not be well explored, leading again to volume underestimation and a potentially suboptimal design center. No theoretical guarantees can be given, which is why we recommend starting several *L*_*p*_-Adaptation runs from different starting points and with different *p* in order to gain an impression of the overall shape of the feasible region.

Rigorous analysis of the algorithmic complexity and solution quality of design-centering and volume-approximation schemes hinges on the convexity of the feasible region^38, 46^, which is an unrealistic assumption in practice and not the intended application domain for *L*_*p*_-Adaptation. Nevertheless, because *L*_*p*_-Adaptation can be understood as a simultaneous rounding and volume-computation scheme^38^, we expect the number of function evaluations that are required to reach a certain level of accuracy to scale polynomially with problem dimensionality. This is confirmed by the empirical scaling shown in Suppl. Fig. 11. Considering arbitrary, non-convex feasible regions, future theoretical analysis of *L*_*p*_-Adaptation might be possible in the PAC (Probably Approximately Correct) framework^47^, which has previously been successfully applied to biological and bio-inspired algorithms. This, however, is beyond the scope of our present work.

Notwithstanding these open questions, the benchmarks presented here advance the state of the art in general design centering and volume estimation. Versatile default parameters and the availability of open-source implementations render *L*_*p*_-Adaptation practically useful, and we expect a number of engineering and biological problems, including novel designs of synthetic biological circuits^3, 48^, to benefit from a re-interpretation in the design-centering framework.

### Data availability statement

A Matlab implementation of *L*_*p*_-Adaptation is available for free download on the MOSAIC Group’s web page at mosaic.mpi-cbg.de and on GitHub at https://github.com/Joe1909/LpAdaptation_Code. The data used in this paper and the scripts to reproduce the figures are available from https://git.mpi-cbg.de/asmus/LpAdaptation_DataPaper.

## Electronic supplementary material

Supplementary Information

## References

[1] Graeb, H. E. *Analog Design Centering and Sizing* (Springer, 2007).

[2] Barnes CP, Silk D, Sheng X, Stumpf MP (2011). Bayesian design of synthetic biological systems. Proceedings of the National Academy of Sciences.

[3] Woods, M. L., Leon, M., Perez-Carrasco, R. & Barnes, C. P. A statistical approach reveals designs for the most robust stochastic gene oscillators. *ACS synthetic biology* (2016). 10.1021/acssynbio.5b00179 PMC4914944 26835539

[4] Grötschel, M., Lovász, L. & Schrijver, A. Geometric algorithms and combinatorial optimization. *Journal of the Operational Research Society* 797 (1988).

[5] Thach PT (1988). The design centering problem as a D.C. programming problem. Mathematical Programming.

[6] Bárány I, Füredi Z (1987). Computing the volume is difficult. Discrete & Computational Geometry.

[7] Khachiyan LG (1989). The problem of calculating the volume of a polyhedron is enumerably hard. Russian Mathematical Surveys.

[8] Seifi A, Ponnambalam K, Vlach J (1999). A unified approach to statistical design centering of integrated circuits with correlated parameters. Circuits and Systems I: Fundamental Theory and Applications, IEEE Transactions on.

[9] Kjellström G, Taxen L (1981). Stochastic optimization in system design. IEEE Trans. Circ. and Syst..

[10] Sapatnekar SS, Vaidya PM, Kang S-M (1994). Convexity-based algorithms for design centering. IEEE Transactions on Computer-Aided Design of Integrated Circuits and Systems.

[11] Director SW, Hachtel GD (1977). The simplicial approximation approach to design centering. Circuits and Systems, IEEE Transactions on.

[12] Vaidya, P. M. A new algorithm for minimizing convex functions over convex sets. In *Foundations of Computer Science, 1989. 30th Annual Symposium on*, 338–343 (IEEE, 1989).

[13] Abdel-Malek HL, Hassan A-KS (1991). The ellipsoidal technique for design centering and region approximation. Computer-Aided Design of Integrated Circuits and Systems, IEEE Transactions on.

[14] Gu, C. & Roychowdhury, J. Yield estimation by computing probabilistic hypervolumes. In *Extreme Statistics in Nanoscale Memory Design*, 137–177 (Springer, 2010).

[15] Storn R (1999). System design by constraint adaptation and differential evolution. IEEE Transactions on Evolutionary Computation.

[16] Tan HK, Ibrahim Y (1999). Design centering using momentum based CoG. Engineering Optimization.

[17] Cousins B, Vempala S (2016). A practical volume algorithm. Mathematical Programming Computation.

[18] Kitano H (2004). Biological robustness. Nature Rev. Genetics.

[19] Dayarian A, Chaves M, Sontag ED, Sengupta AM (2009). Shape, size, and robustness: feasible regions in the parameter space of biochemical networks. PLoS Comput Biol.

[20] von Dassow G, Meir E, Munro EM, Odell GM (2000). The segment polarity network is a robust developmental module. Nature.

[21] Beyer H-G, Schwefel H-P (2002). Evolution strategies–a comprehensive introduction. Natural computing.

[22] Whitley D (1994). A genetic algorithm tutorial. Statistics and Computing.

[23] Hafner M, Koeppl H, Hasler M, Wagner A (2009). ‘Glocal’ robustness analysis and model discrimination for circadian oscillators. PLoS Comput. Biol..

[24] Singhal K, Pinel J (1981). Statistical design centering and tolerancing using parametric sampling. IEEE Transactions on Circuits and Systems.

[25] Schwencker, R., Schenkel, F., Graeb, H. & Antreich, K. The generalized boundary curve - a common method for automatic nominal design centering of analog circuits. In *Proceedings of the Conference on Design, Automation and Test in Europe*, DATE’ 00, 42–47 (ACM, New York, NY, USA, 2000).

[26] Soin, R. & Spence, R. Statistical exploration approach to design centring. In *IEE Proceedings G (Electronic Circuits and Systems)*, vol. 127, 260–269 (IET, 1980).

[27] Harnisch T, Kunert J, Toepfer H, Uhlmann H (1997). Design centering methods for yield optimization of cryoelectronic circuits. IEEE transactions on applied superconductivity.

[28] Vidigal LM, Director SW (1982). A design centering algorithm for nonconvex regions of acceptability. IEEE Transactions on Computer-Aided Design of Integrated Circuits and Systems.

[29] Hassan, A.-K. S. & Mohamed, A. S. Surrogate-based circuit design centering. In *Surrogate-Based Modeling and Optimization*, 27–49 (Springer, 2013).

[30] Storn R, Price K (1997). Differential evolution – a simple and efficient heuristic for global optimization over continuous spaces. Journal of Global Optimization.

[31] González, I. & Sánchez, I. Optimal centering and tolerance design for correlated variables. *The International Journal of Advanced Manufacturing Technology* 1–12 (2013).

[32] González, I. & Sánchez, I. Optimal centering and tolerance synthesis for non-independent and non-normal variables. *International Journal of Advanced Manufacturing Technology* **79** (2015).

[33] Hansen N, Ostermeier A (2001). Completely derandomized self-adaptation in evolution strategies. Evol. Comput..

[34] Müller, C. L. & Sbalzarini, I. F. Gaussian Adaptation revisited — an entropic view on covariance matrix adaptation. In *Proc. EvoStar, vol. 6024 of Lect. Notes Comput. Sci*., 432–441 (Springer, Istanbul, Turkey, 2010).

[35] Müller, C. L. & Sbalzarini, I. F. Gaussian Adaptation as a unifying framework for continuous black-box optimization and adaptive Monte Carlo sampling. In *Proc. IEEE Congress on Evolutionary Computation (CEC)*, 2594–2601 (Barcelona, Spain, 2010).

[36] Müller, C. L. & Sbalzarini, I. F. Gaussian Adaptation for robust design centering. In (eds) Poloni, C., Quagliarella, D., Périaux, J., Gauger, N. & Giannakoglou, K. *Evolutionary and deterministic methods for design, optimization and control, Proc. EuroGen*, 736–742 (CIRA, ECCOMAS, ERCOFTAC, Capua, Italy, 2011).

[37] Hansen, N. Adaptive encoding for optimization. *Research Report 6518*, INRIA (2008).

[38] Simonovits M (2003). How to compute the volume in high dimension?. Mathematical programming.

[39] Vempala, S. S. Recent progress and open problems in algorithmic convex geometry. In *LIPIcs-Leibniz International Proceedings in Informatics*, vol. 8 (Schloss Dagstuhl-Leibniz-Zentrum fuer Informatik, 2010).

[40] Lasserre, J. Unit balls of constant volume: which one has optimal representation? *arXiv preprint arXiv:1408.1324* (2014).

[41] Henrion D, Lasserre JB, Savorgnan C (2009). Approximate volume and integration for basic semialgebraic sets. SIAM review.

[42] Gruber PM (2011). John and Loewner ellipsoids. Discrete & Computational Geometry.

[43] Kim J-R, Cho K-H (2006). The multi-step phosphorelay mechanism of unorthodox two-component systems in E. coli realizes ultrasensitivity to stimuli while maintaining robustness to noises. Computational biology and chemistry.

[44] Zamora-Sillero E, Hafner M, Ibig A, Stelling J, Wagner A (2011). Efficient characterization of high-dimensional parameter spaces for systems biology. BMC systems biology.

[45] Toni T, Welch D, Strelkowa N, Ipsen A, Stumpf MPH (2009). Approximate Bayesian computation scheme for parameter inference and model selection in dynamical systems. J. R. Soc. Interface.

[46] Lovász L, Vempala S (2006). Simulated annealing in convex bodies and an O*(N4) volume algorithm. Journal of Computer and System Sciences.

[47] Valiant, L. *Probably Approximately Correct: Nature’s Algorithms for Learning and Prospering in a Complex World* (Basic Books, Inc., New York, NY, USA, 2013).

[48] Hold, C., Billerbeck, S. & Panke, S. Forward design of a complex enzyme cascade reaction. *Nature Communications* **7** (2016). 10.1038/ncomms12971 PMC5052792 27677244

