# Supplementary material for: Lp-Adaptation: Simultaneous Design Centering and Robustness Estimation of Electronic and Biological Systems
Source: Sci Rep. 2017 Jul 27;7:6660. doi: 10.1038/s41598-017-03556-5 (PMC5532288; doi:10.1038/s41598-017-03556-5)
Supplement: Supplementary file 1 — Supplementary Information [file 41598_2017_3556_MOESM1_ESM.pdf]

L_p-Adaptation: Simultaneous Design Centering and Robustness Estimation of Electronic and Biological Systems
— Supplementary Information —

Josefine Asmus[1,2,3], **Christian L. Müller**[4,*], **and Ivo F. Sbalzarini**[1,2,3,5,**]

[1]Chair of Scientific Computing for Systems Biology, Faculty of Computer Science, TU Dresden, 01069 Dresden, Germany
[2]Center for Advancing Electronics Dresden (cfaed), TU Dresden, 01069 Dresden, Germany
[3]MOSAIC Group, Center for Systems Biology Dresden (CSBD), 01307 Dresden, Germany
[4]Simons Center for Data Analysis, Simons Foundation, New York, 10010, USA
[5]Max Planck Institute of Molecular Cell Biology and Genetics, 01307 Dresden, Germany
[*]cmueller@simonsfoundation.org
[**]ivos@mpi-cbg.de

Supplementary Note 1: The L_p-Adaptation Algorithm

We provide here the details of the L_p-Adaptation algorithm, an adaptive statistical method for approximate design centering and volume estimation. L_p-Adaptation is inspired by Gaussian Adaptation (GaA)[1,2], but instead of sampling from a Gaussian proposal distribution, we sample uniformly from L_p-balls, as detailed in Algorithm 1. The choice of the L_p-ball reflects assumptions about the noise performance of the systems parameters. For example, the L_2-ball assumes a Gaussian perturbation prior, L_∞ the worst-case scenario. Inspired by the covariance matrix adaptation evolutionary strategy (CMA-ES)[3], we use a multi-sample strategy and update the covariance as described[4]. We also introduce an adaptive schedule for changing the target hitting probability of the sampler in order to trade off the conflicting requirements of design centering (increased hitting probability) and volume approximation (decreased hitting probability). As depicted in Supplementary Figure 1, L_p-Adaptation successively adapts the proposal distribution to the unknown feasible region. The complete pseudo-code is given in Algorithm 2.

L_p-Adaptation can be interpreted as a synthetic evolutionary process that maximizes the robustness, rather than the fitness, of the underlying system. Robustness is measured in terms of the volume $\mathrm{vol}(L_p^n)$ of an n-dimensional L_p-ball, of which a certain fraction P (i.e., the target hitting probability) overlaps with the feasible region A. Let us denote by $\mathscr{L}_p^n = \{\mathbf{m}, \mathbf{C}\}$ the set of all n-dimensional L_p-balls where $\mathbf{m} \in \mathbb{R}^n$ denotes the center of the L_p-ball, and $\mathbf{C} \in \mathscr{S}_+^{n\times n}$ is a symmetric positive-definite (covariance) matrix defining the affine map for scaling and rotation of the L_p-ball. L_p-Adaptation then seeks to maximize the following robustness criterion:

$$\begin{aligned} \max_{L_p^n \in \mathscr{L}_p^n} \quad & \mathrm{vol}(L_p^n) \\ \text{s.t.} \quad & \mathbf{m} \in A \\ & \tfrac{\mathrm{vol}(A \cap L_p^n)}{\mathrm{vol}(L_p^n)} \geq P\,. \end{aligned} \tag{1}$$

The volume of an L_p-ball is completely determined by the volume of the unit L_p-ball (with zero mean and n-dimensional identity matrix $\mathbf{C} = \mathbf{1}$) and the determinant of the matrix $\mathbf{C}$. Thus, the robustness criterion can be rewritten as a non-convex log-det maximization problem:

$$\begin{aligned} \max_{L_p^n \in \mathscr{L}_p^n} \quad & \log\det \mathbf{C} \\ \text{s.t.} \quad & \mathbf{m} \in A \\ & \tfrac{\mathrm{vol}(A \cap L_p^n)}{\mathrm{vol}(L_p^n)} \geq P\,. \end{aligned} \tag{2}$$

This objective function provides a natural non-convex extension of the maximum inscribed ellipsoid method of Seifi *et al.*[5]. For instance, if A is a convex polyhedron with known parameterization and $P = 1$, then Problem 2 is a convex problem that can be efficiently solved using interior point methods. However, in the general case, no efficient algorithms exist to solve Problem 2. L_p-Adaptation approximately solves this problem by using a synthetic evolutionary process consisting of the following four steps: **Initialization**, **Sampling**, **Evaluation**, and **Adaptation**, which are repeated in iterations until a stopping criterion is fulfilled (e.g., a maximal number of evaluations of the specifications is reached). The L_p-Adaptation algorithm requires two inputs: (1) a feasible starting point, and (2) a membership oracle that checks whether any given point is feasible or not by evaluating the specifications. All other algorithm parameters have default values as given in Algorithm 2 and do not necessarily need to be set by the user. Below, we discuss the individual steps of the algorithm in detail.

Initialization

The goal of initialization is to determine the initial shape of the proposal distribution. Therefore, the initial feasible point is used as the mean of the initial proposal distribution. Since the proposal is an L_p-ball, it is completely determined by:

1. the norm $p > 0$,
2. the ball radius $r \in \mathbb{R}^+$, and
3. an affine transformation matrix $\mathbf{C} = r^2(\mathbf{Q})(\mathbf{Q})^T$,
   where $\mathbf{Q} \in \mathbb{R}^{n\times n}$ and $\det \mathbf{Q} = 1$.

The norm p can be given by the user and never changes throughout the algorithm. $\mathbf{Q}$ and r are dynamically adapted, starting from the initial values $\mathbf{C} = \mathbf{I}$ and $r = 1$. The sample size per iteration $\lambda \in \mathbb{N}^+$ and the target hitting probability $P \in [0,1]$ are also initialized automatically to $P = \frac{1}{\mathrm{e}}$ (information-theoretic optimum for Gaussian proposals[1]) and $\lambda = 4 + \lfloor 3 * \log(n) \rfloor$ (default for CMA-ES[3]).

Sampling

Sampling generates candidate solutions by drawing λ random numbers uniformly distributed in the proposal L_p-ball. This is efficiently done by first sampling points in the unit L_p-ball in n dimensions, as detailed in Algorithm 1, and then transforming the samples using the affine map $\mathbf{Q}$ and scaling them to radius r. This is detailed in Algorithm 2, Line 12, and it samples λ points from the current, adapted proposal distribution.

Evaluation

For each of the λ points, the algorithm needs to evaluate whether it is feasible or not. For this binary decision, the provided membership oracle is queried for all points, internally checking them against the specifications. The number of feasible points is called μ.

Adaptation

Using the information gained during Evaluation, the algorithm adapts the proposal in order to find the largest proposal with the given hitting probability. This is illustrated in Supplementary Figure 2, showing two proposals that have the same hitting probability, but differ in size. In order to work toward larger sizes, $\mathbf{Q}$ and r are adapted in each iteration such that the hitting probability remains constant. Radius, mean, and covariance of the affine map are adapted as detailed below.

radius

The proposal radius r is adapted as shown in Algorithm 2, Line 15, where $f_e > 1$ is an expansion factor and $f_c < 1$ is a contraction factor. The factors f_e and f_c are chosen such that the hitting probability remains constant under stationary conditions, which means that the volume of the proposal distribution

$$\det(\mathbf{C}) = r^{2n}\det(\mathbf{Q}\mathbf{Q}^T) \tag{3}$$

does not change[1]. For each feasible point, the radius is increased by a factor f_e, and for each infeasible point it is decreased by a factor f_c. Overall, this changes the proposal volume by factors of f_e^{2n} and f_c^{2n}, respectively. Assume there are S feasible and F infeasible points at stationarity. Since the total volume must not change, this leads to the condition

$$\prod_{i=1}^{S}(f_e)^{2n}\prod_{i=1}^{F}(f_c)^{2n} = 1. \tag{4}$$

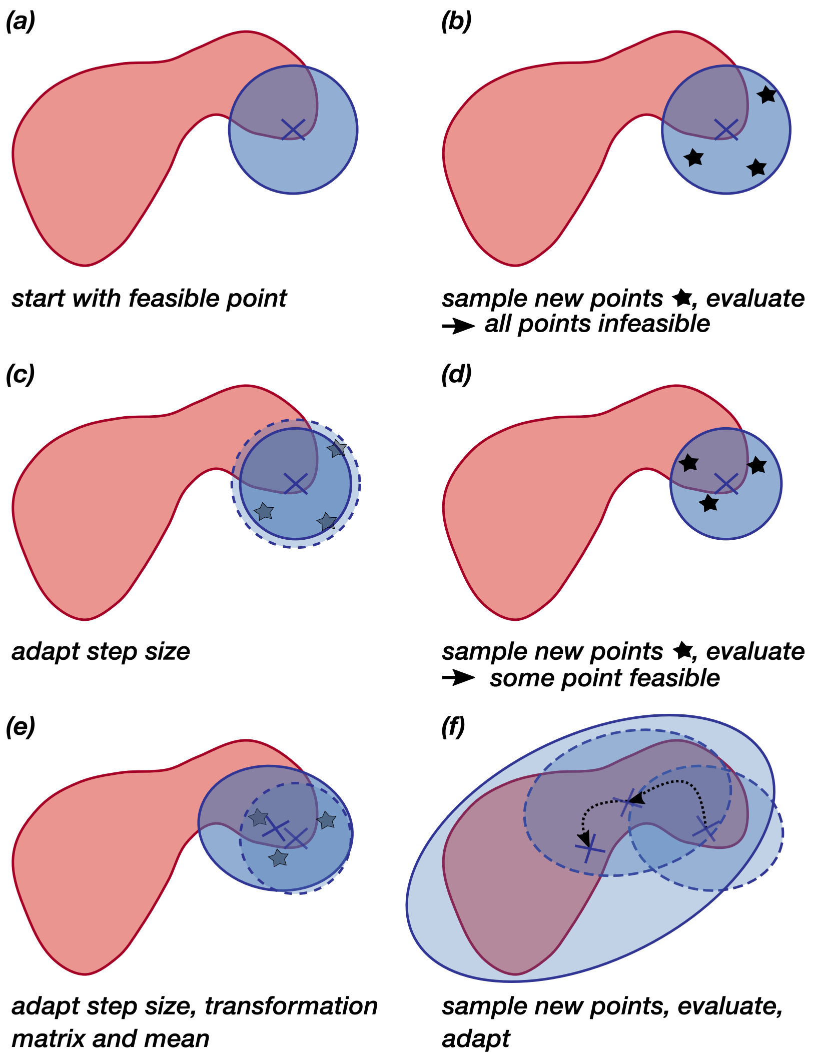

Supplementary Figure 1. Schematic illustration of the algorithmic procedure of adapting the proposal distribution (blue ellipse) to the feasible region (red). The cross represents the mean of the proposal distribution. (a) The algorithm requires a feasible point so start with. This feasible point is set as the mean (center) of the initial proposal distribution. The initial proposal ball is isotropic with a radius of 1. (b,d) New points are drawn from the proposal and evaluated against the specifications by querying the membership oracle. (c) If all points are infeasible, the ball radius is reduced in order to increase the probability of sampling a feasible point next. The shape of the proposal remains unchanged. (e) If at least one point is feasible, the location, shape, and radius of the proposal are adapted by moving the mean in the direction of the center of all feasible points, increasing the radius, and adapting the affine transformation to include information about the distribution of feasible points. (f) At the end of the process, the proposal will have the largest possible volume for the given target hitting probability. Now, the mean can be used as a design center, and the volume can be approximated from the determinant of the proposal.

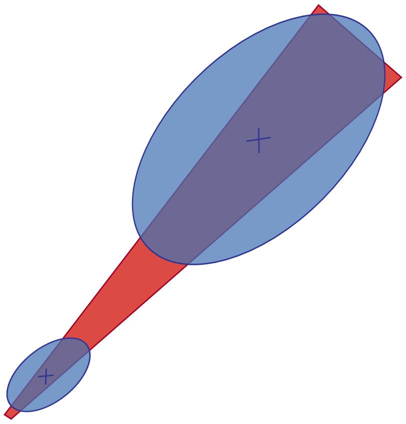

Supplementary Figure 2. The two proposal distributions (blue) have the same hitting probability over the red feasible region. The adaptation scheme increases the size (volume) of the proposal under constant hitting probability.

Since the effective, empirical hitting probability, P_{emp}, at stationarity is equal to the target hitting probability, i.e., the algorithm has converged, and assuming f_e and f_c to be near 1, we get:

$$f_e = 1 + \beta(1 - P) \tag{5}$$

$$f_c = 1 - \beta P, \tag{6}$$

where P is the target hitting probability, and the learning rate $\beta \in [0,1]$ regulates the speed of adaptation. Too large a β leads to oscillations in the radius; too small a β leads to slow adaptation. We use the default value

$$\beta = \frac{0.6}{(n+1.3)^2 + P\lambda}. \tag{7}$$

mean

If at least one feasible point was sampled, i.e., $\mu > 0$, the mean of the proposal is adapted according to:

$$\mathbf{m} \leftarrow (1 - c_m)\mathbf{m} + c_m \frac{1}{\mu}\mathbf{X}\mathbf{b}\,, \tag{8}$$

where $\mathbf{X}_{:,j}$ is the j-th sampled point, and b_j is a binary variable indicating the feasibility of $\mathbf{X}_{:,j}$ (1: feasible, 0: infeasible). The parameter $0 \leq c_m \leq 1$ defines how far the mean is moved in the direction of the center of mass of the new feasible points. If $c_m = 0$, the mean is not adapted at all, if $c_m = 1$, the mean immediately jumps to the center of mass of the μ new feasible points. We propose to use $c_m = 1/(\mathrm{e}n)$[6]. It is also possible to make c_m depend on the number of feasible points per iteration, as $c_m = \min(\frac{\mu}{\mathrm{e}n}, 0.5)$, such that the mean moves faster if more feasible points are found.

covariance

If at least one feasible point was sampled, the covariance of the affine mapping of the proposal is adapted according to:

$$\mathbf{C} \leftarrow (1 - c_1 - c_\mu)\mathbf{C} + c_1 \mathbf{p_c}\mathbf{p_c}^T + c_\mu \mathbf{C}_\mu\,, \tag{9}$$

where the second term is a rank-one update proportional to the difference between the new and the old mean. The third term is a rank-μ update, adding μ linearly independent directions into the matrix, i.e., the information in the μ directions learned from the feasible samples. The details of these updates are as previously described[4]. The learning rates $c_1 \in [0,1]$ and $c_\mu \in [0,1]$ determine the weights of the rank-one update and the rank-μ update, respectively. Their sum must be smaller than 1. As suggested previously[4], we set

$$c_1 = \alpha_c \frac{0.2}{(n+1.3)^2 + \mu} \tag{10}$$

and

$$c_\mu = 0.2\alpha_c \frac{\mu - 2 + \frac{1}{\mu}}{(n+2)^2 + \alpha_\mu \mu}, \tag{11}$$

where $\alpha_c = 3$ and $\alpha_\mu = 0.2$ were found in a parameter study. Both c_1 and c_μ depend on the number of feasible points in one iteration μ, such that larger μ give more importance to the rank-μ update.

Algorithm 1: Uniformly sampling from a unit L_p-ball, see Section 4.1 in Ref.[7]

Input : dimension n, $p > 0$
Output : real random vector y uniformly distributed in $L_p^n(1)$

1. Sample n real scalars *i.i.d.* from the generalized Gamma distribution $\xi_i \sim \tilde{G}(\frac{1}{p}, p)$.
2. Construct a vector $\mathbf{x} \in \mathbb{R}^n$ with components $x_i = s_i \xi_i$, where s_i are independent uniformly random signs.
3. Compute $z = w^{1/n}$, where w is a random variable uniformly distributed in the interval $[0, 1]$.
4. Return $\mathbf{y} = z \frac{\mathbf{x}}{\|\mathbf{x}\|_p}$, where $\|\mathbf{x}\|_p = (\sum_{i=1}^n |x_i|^p)^{1/p}$.

Adapting the Hitting Probability

Introducing a schedule for changing the hitting probability makes it possible to find a better design center or obtain a better volume approximation. The sequence of target hitting probabilities can be given as an input to the algorithm.

It should start from a value around $\frac{1}{e}$ in order to first learn the location and the approximate shape of the feasible region. This information is then used to perform "warm starts" with the subsequent target hitting probabilities. This changes the target hitting probability according to a **fixed, predefined schedule**. Alternatively, a **variable schedule** can be used when *lowering* the hitting probability for volume estimation.

For the fixed schedule, the user defines in advance after how many function evaluations the hitting probability is changed, and to what values. For the variable schedule, the hitting probability is decreased to predefined values whenever the process is converging. This is decided by looking at the relative changes if the radius r, the empirical hitting probability P_{emp}, and the volumes of the axes-aligned bounding box V_{BB} and the Loewner ellipsoid V_L of all feasible points. The Loewner ellipsoid[8]of a set of points is the unique minimal-volume ellipsoid that contains this set of points. To approximate this ellipsoid we use Anye Li's MATLAB implementation of Khachiyan's algorithm[9]. If r, P, V_{BB}, and V_L are not changing anymore, we assume the proposal distribution "has seen" all of the feasible region that is possible to see with the current hitting probability. Then, the target hitting probability is lowered. If lowering the hitting probability does not lead to a larger estimated volume, we assume that the entire feasible region has been covered by the proposal and stop the algorithm.

Supplementary Note 2: 2D Benchmarks for Volume Estimation

We benchmark volume estimation using two standard 2D test problems from the literature: the "Handle"[10] and the "Folium"[11]. Supplementary Figures 3 and 4 show the averages and the standard deviations of the approximated volumes of the two regions versus the number of function evaluations for four different L_p-balls. The results are compared with those from uniform sampling ("bruteforce", gray diamonds) and with the upper bounds provided by Loewner ellipsoids[8] (blue circles) and the axes-aligned bounding box (green squares) of all feasible points. The true volume is indicated by the dashed black line. Similar to what we observed for $p = 2$ in the main text, L_p-Adaptation provides a good volume approximation in all cases. Not only the L_p-ball with $p = 2$, but also with $p = 0.5$, 1, and ∞ lead to good results. Initially the radius of all L_p-balls is set to 1. Therefore, the first volume approximation is larger the higher the chosen p-norm of the L_p-ball. As long as the volumes of axis-aligned bounding box and Loewner ellipsoid are increasing, new points in the corners of the test bodies are found. We conclude that the choice of p-norm for the proposal is irrelevant in these low-dimensional examples and that the estimation accuracy of L_p-Adaptation is comparable with that of brute-force sampling.

Supplementary Note 3: Switched Capacitor Filter

Design centering of a Switched-Capacitor (SC) filter pulse-code modulator (PCM) with parasitic capacitances has been introduced by Storn[12] as a test case for design-centering algorithms. The transfer function of the SC-PCM filter is:

$$H(f) = \frac{V_{\text{out}}}{V_{\text{in}}} = H_1(f) \cdot H_2(w) \cdot H_3(w) \tag{12}$$

with

$$w = j \cdot \tan \frac{\pi f}{f_a} \tag{13}$$

Algorithm 2: L_p-Adaptation for approximate design centering and volume estimation with L_p-balls

Input : Initial feasible point $\mathbf{m}^{(0)} \in \mathbb{R}^n$
membership oracle $f : \mathbb{R}^n \to \{0,1\}$ (check specifications)

Output : Design center $\mathbf{m}^{(K)}$, $\mathbf{Q}^{(K)}$, radius $r^{(K)}$, empirical hitting probability $P_{\text{emp}}^{(K)}$

Initialize : transformation matrix $\mathbf{C}^{(0)} \in \mathbb{R}^{n \times n}$, default: $\mathbf{I}$
rank-one update vector $\mathbf{p_c} \in \mathbb{R}^n, \mathbf{p_c} = \mathbf{0}$
p-norm $p > 0$, default $p = 2$
population size $\lambda \in \mathbb{N}^+$, default: $4 + \lfloor 3\log(n) \rfloor$
weighting factor $c_m \in [0,1]$, default: $1/(en)$
learning constant for rank-one update c_p, default: $\frac{1}{\sqrt{n}}$
window size for P_{emp} $w \in \mathbb{N}^+$, such that $\frac{w}{\lambda} \in \mathbb{N}^+$, default: $\lfloor \frac{300}{\lambda} \rfloor \lambda$
vector of target hitting probabilities $\mathbf{P}$, where $\mathbf{P}_{l,l=1\cdot m} \in [0,1]$, default: 0.35
# of iterations $\mathbf{K}$, where $\mathbf{K}_{l,l=1,\cdots,m} \in \mathbb{N}^+$, $\mathbf{K}_0 = 0$, such that $\mathbf{K}_l - \mathbf{K}_{l-1}$ iterations with $\mathbf{P}_l$, default: $\lfloor \frac{1000}{\lambda} \rfloor n$

$\mathbf{BDDB} \leftarrow \mathbf{C}^{(0)}$ /* Eigen-decomposition, $\mathbf{B}$ orthogonal, $\mathbf{D}$ diagonal with elements sorted ascendingly */
$\mathbf{Q} \leftarrow \mathbf{BD}$
$\mathbf{Q}^{(0)} \leftarrow \frac{1}{(\det \mathbf{Q})^{1/n}} \mathbf{Q}$ // normalize $\mathbf{Q}$, such that $\det \mathbf{Q} = 1$
$r^{(0)} \leftarrow \sqrt[2n]{\det \mathbf{C}^{(0)}}$ // $\mathbf{C} = r^2 \mathbf{Q}\mathbf{Q}^T$, all volume information in step size r
for $l \leftarrow 1$ **to** m **do**
  set learning rate $\beta \in [0,1]$, default: $\frac{0.6}{(n+1.3)^2 + \mathbf{P}_l \lambda}$
  $f_e \leftarrow 1 + \beta(1 - \mathbf{P}_l)$ // expansion factor
  $f_c \leftarrow 1 - \beta \mathbf{P}_l$ // contraction factor
  for $g \leftarrow \mathbf{K}_{l-1} + 1$ **to** $\mathbf{K}_l$ **do**
    /* λ samples uniformly from L_p-ball with radius r, centered at $\mathbf{m}$, deformed by $\mathbf{C}$ */
    for $j \leftarrow 1$ **to** λ **do**
      Sample $\mathbf{y} \sim L_p^n(1)$ // sample uniformly from L_p-ball with radius 1, see Algorithm 1
      $\mathbf{X}_{:,j}^{(g)} \leftarrow \mathbf{m}^{(g-1)} + r^{(g-1)} (\mathbf{Q}^{(g-1)} \mathbf{y})$ // affine transform of samples
      Evaluate $b_j = f(\mathbf{X}_{:,j}^{(g)})$ // if $\mathbf{X}_{:,j}^{(g)}$ feasible, $\mathbf{b}_j = 1$, else $\mathbf{b}_j = 0$
    count number of feasible points μ
    Adapt $r^{(g)} \leftarrow f_e^{\mu} \cdot f_c^{\lambda - \mu} r^{(g-1)}$ // adapt ball radius
    if $\mu > 0$ **then**
      Adapt $\mathbf{m}^{(g)} \leftarrow (1 - c_m)\mathbf{m}^{(g-1)} + c_m \cdot \frac{1}{\mu}(\mathbf{X}^{(g)} \cdot \mathbf{b})$ // adapt mean
      set scalars $\alpha_j \geq 0$, for $j = 0, \ldots, \mu$ // α_j normalize input entries for matrix C
      $\mathbf{p_c} \leftarrow (1 - c_p)\mathbf{p_c} + \sqrt{c_p(2 - c_p)}\alpha_0(\mathbf{m}^{(g)} - \mathbf{m}^{(g-1)})$ // rank-one update
      $\mathbf{C}_\mu \leftarrow \sum_{j=1}^{\lambda} \mathbf{b}_j \frac{1}{\mu} \alpha_j^2 (\mathbf{X}_{:,j}^{(g)} - \mathbf{m}^{(g-1)})(\mathbf{X}_{:,j}^{(g)} - \mathbf{m}^{(g-1)})^T$ // use all feasible points for rank-μ update
      compute learning rates $c_1 \in [0,1]$ for rank-1 update, and $c_\mu \in [0,1]$ for rank-μ update, see equations 10 and 11
      Adapt $\mathbf{C}^{(g)} \leftarrow (1 - c_1 - c_\mu)\mathbf{C}^{(g-1)} + c_1 \mathbf{p_c} \mathbf{p_c}^T + c_\mu \mathbf{C}_\mu$ // adapt affine transformation matrix
      $\mathbf{BDDB} \leftarrow \mathbf{C}^{(g)}$ // Eigen-decomposition
      $\mathbf{Q} \leftarrow \mathbf{BD}$
      $\mathbf{Q}^{(g)} \leftarrow \frac{1}{(\det \mathbf{Q})^{1/n}} \mathbf{Q}$ // normalize $\mathbf{Q}$, such that $\det \mathbf{Q} = 1$
    else
      $\mathbf{m}^{(g)} = \mathbf{m}^{(g-1)}$
      $\mathbf{p_c} = (1 - c_p)\mathbf{p_c}$
      $\mathbf{Q}^{(g)} = \mathbf{Q}^{(g-1)}$
      $\mathbf{C}^{(g)} = \mathbf{C}^{(g-1)}$
    $P_{\text{emp}}^{(g)} = \frac{\#\text{ of feasibe points in } \min((g - \mathbf{K}_{l-1})\lambda, w) \text{ previous evaluations}}{(\min((g - \mathbf{K}_{l-1})\lambda, w))}$

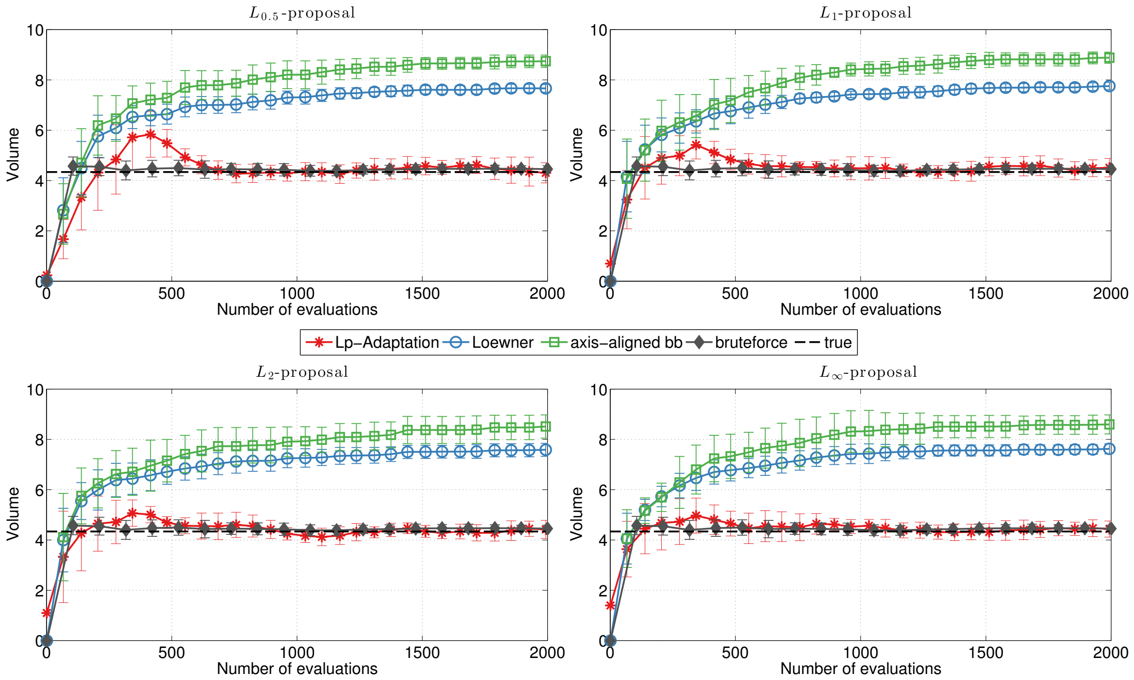

Supplementary Figure 3. Average and standard deviation over ten independent runs of L_p-Adaptation to estimate the volume of the "Handle" (i.e., Lasserre's "Body2"[10]). The results are compared with brute-force sampling. Two different upper bounds are obtained from the standard Loewner ellipsoid and the axis-aligned bounding box of all feasible points. The four panels use different L_p-balls as proposal distributions.

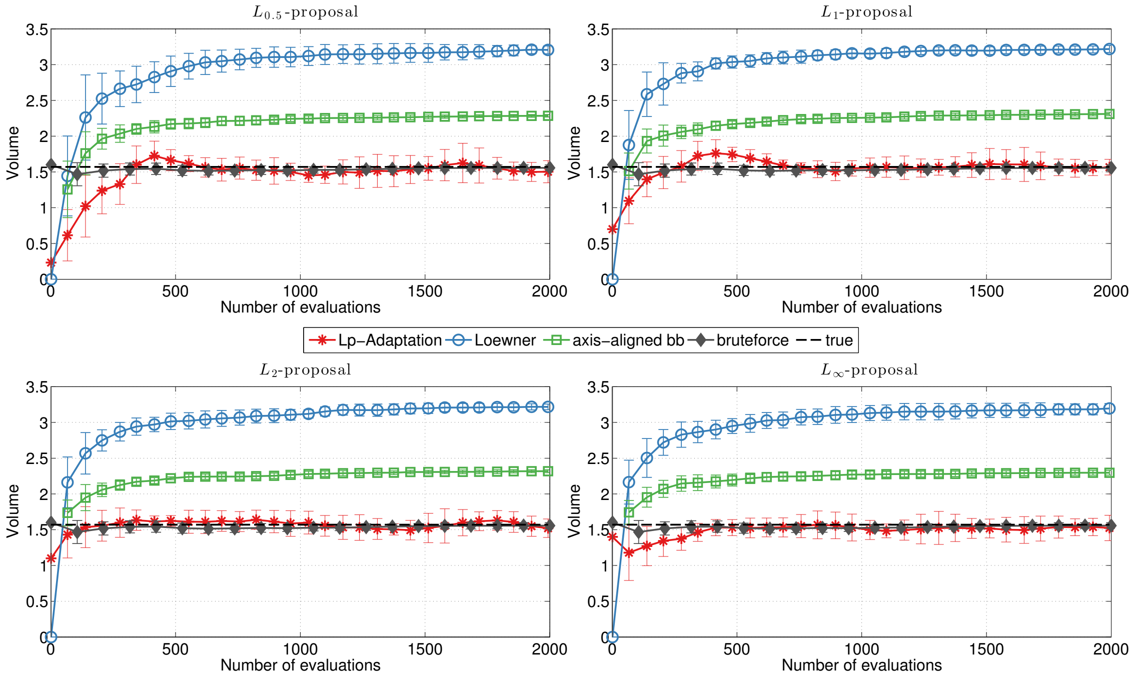

Supplementary Figure 4. Average and standard deviation over ten independent runs of L_p-Adaptation to estimate the volume of the "Folium"[11]. The results are compared with brute-force sampling. Two different upper bounds are obtained from the standard Loewner ellipsoid and the axis-aligned bounding box of all feasible points. The four panels use different L_p-balls as proposal distributions.

and

$$j = \sqrt{-1}, \tag{14}$$

where f_a is the sampling frequency of the filter and f is the signal frequency. The chosen f_a for $H_2(w)$ is 128 kHz, and 32 kHz for $H_3(w)$, as in the original publication[12].

The transfer function of the analog RC lowpass pre-filter is:

$$H_1(f) = \frac{1}{1 + j \cdot 2\pi f \cdot R_0 \cdot C_0} = \frac{1}{1 + j \cdot f \cdot v_1}. \tag{15}$$

For the SC-PCM lowpass filter with parasitic capacitances, the transfer functions $H_2(w)$ and $H_3(w)$ can be written as

$$H_2(w) = \frac{w^2\left[\left(v_{32} - \frac{\gamma}{2}\right)\left(v_{12}(1+\varepsilon) + \frac{\gamma}{2}\right) + v_{132}\frac{\gamma}{2}\right] + w\left[\left(1+\frac{\gamma}{2}\right)(v_{32} - v_{132}) + v_{12}(1+\varepsilon)\left(1+\frac{\gamma}{2}\right)\right] + \left(1+\frac{\gamma}{2}\right)^2}{w^2\left[\left(v_{32} + v_{532} - \frac{\gamma}{2}\right)\left(v_{12}(1+\varepsilon) + \frac{\gamma}{2}\right) + v_{132}\frac{\gamma}{2}\right] + w\left[\left(1+\frac{\gamma}{2}\right)(v_{32} + v_{532} - v_{132}) + v_{12}(1+\varepsilon)\left(1+\frac{\gamma}{2}\right)\right] + \left(1+\frac{\gamma}{2}\right)^2} \tag{16}$$

$$H_3(w) = \frac{w^2\left[\left(v_{33} - \frac{\gamma}{2}\right)\left(v_{13}(1+\varepsilon) + \frac{\gamma}{2}\right) + v_{133}\frac{\gamma}{2}\right] + w\left[\left(1+\frac{\gamma}{2}\right)(v_{33} - v_{133}) + v_{13}(1+\varepsilon)\left(1+\frac{\gamma}{2}\right)\right] + \left(1+\frac{\gamma}{2}\right)^2}{w^2\left[\left(v_{33} + v_{533} - \frac{\gamma}{2}\right)\left(v_{13}(1+\varepsilon) + \frac{\gamma}{2}\right) + v_{133}\frac{\gamma}{2}\right] + w\left[\left(1+\frac{\gamma}{2}\right)(v_{33} + v_{533} - v_{133}) + v_{13}(1+\varepsilon)\left(1+\frac{\gamma}{2}\right)\right] + \left(1+\frac{\gamma}{2}\right)^2}, \tag{17}$$

where the constants $\gamma \in [2.55\%, 10.5\%]$ and $\varepsilon \in [0.1\%, 1\%]$ represent the parasitic effects. We set $\gamma = 5\%$ and $\varepsilon = 0.5\%$, as in the original publication[12].

A filter fulfills the specifications if the magnitude of its transfer function, $|H(f)|$ is above $\{1.0, 1.0292, 1.0, 0.031623\}$ at frequencies $\{0, 200, 3600, 4600\}$Hz and below $\{0.0, 0.97162, 0.94951, 0.90157, 0.0\}$ at frequencies $\{0, 300, 2400, 3000, 3400\}$Hz.

Supplementary Figure 5 shows a comparison of the robustness of the design centers found by L_p-Adaptation with those reported by Storn *et al.*[12]. We measure robustness by the size (radius) of a hyper-cube or hyper-ellipsoid that contains a given fraction of all feasible points, as discussed in the main text. In all cases, the design centers found by L_p-Adaptation are more robust than the previous results, as indicated by the larger radii for all target hitting probabilities.

Supplementary Note 4: Bacterial Two-Component System

For design centering and robustness quantification of biological networks, models of two flavors of the bacterial two-component system (TCS) have been proposed[13,14]. Both chemical reaction networks are modeled as a system of ordinary differential equations. The design parameters are the kinetic rate constants of the reactions, which are to be chosen such that the network fulfills a certain function and is robust against fluctuations in the reaction rates. In order to evaluate the specifications, we numerically solve the differential equations over the time interval $0 \le t \le 10$. All variables are in the range $[0, 1000]$. The two systems differ in their complexity (i.e., number of design parameters) and are called the "orthodox system" and the "unorthodox system", respectively, as described below. All simulations are done in MATLAB 8.2.0 (R2013b) using the "Systems Biology Toolbox 2" and the "SBPD Extension Package"[15] with a time step size of 0.001 until final time 10.

Orthodox system

The model assumes that the total concentrations of the Histidine Kinase (HK) and Response Regulator (RR) proteins sum up to 1, hence:

$$HK + HK_p = 1$$
$$RR + RR_p = 1,$$

where a subscript p denotes the phosphorylated form of the molecule. This leads to the following model equations for the time dynamics of the concentrations $[\cdot]$ of all involved chemical species:

$$\frac{d[HK]}{dt} = k_2(1 - [HK])(1 - [RR_p]) + k_3(1 - [HK]) - k_4[HK][RR_p] - k_1[HK][S]$$
$$\frac{d[RR_p]}{dt} = k_2(1 - [HK])(1 - [RR_p]) - k_4[HK][RR_p] - k_5[RR_p].$$

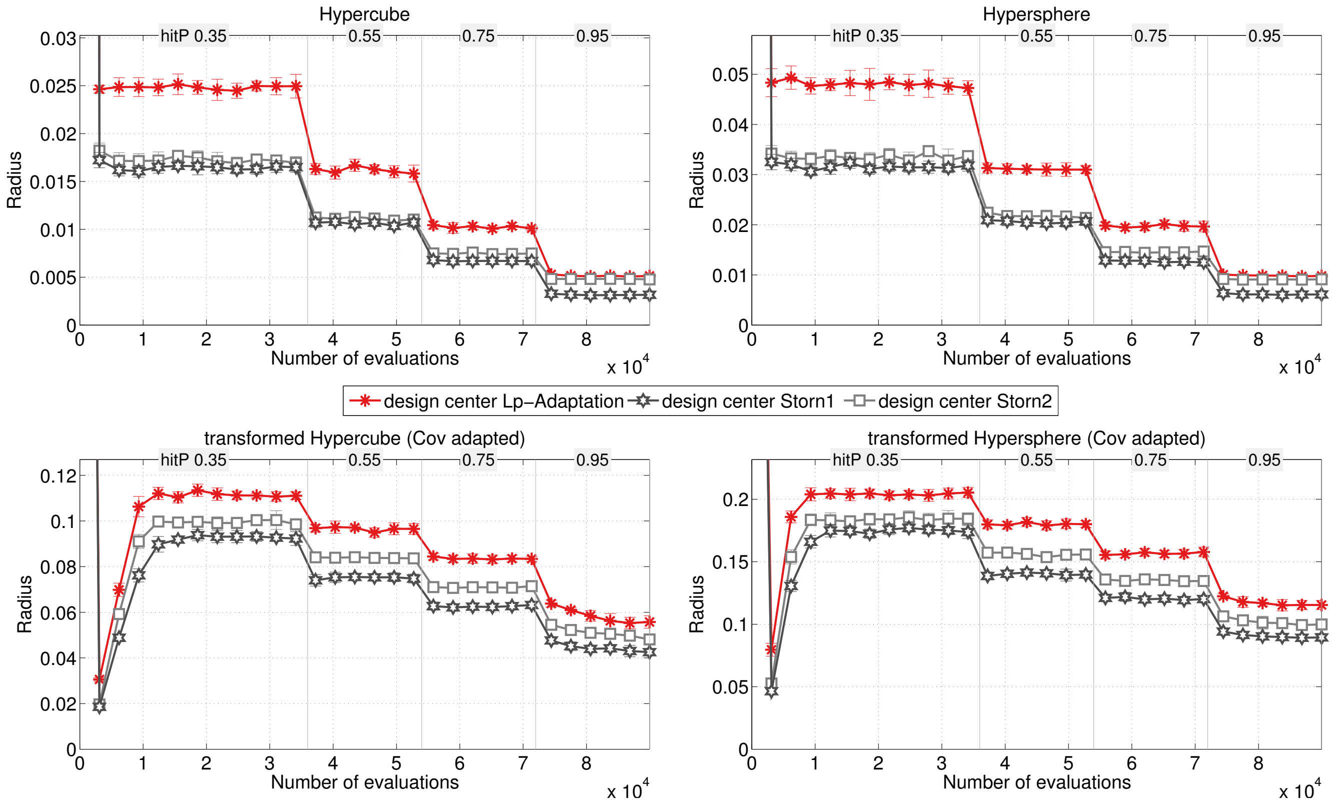

Supplementary Figure 5. Comparison of the robustness of three different design centers: one found by L_p-Adaptation (red star) and two reported by Storn[12] (dark-gray asterisk and light-gray square). We run L_p-Adaptation for these different design centers with a fixed mean and a decreasing schedule of the hitting probability as shown at the top of each plot. In the top row we show the results for the runs with fixed covariances (identity matrix), in the bottom row we allow covariance adaptation. We test two proposals: L_∞ (left, hyper-cube) and L_2 (right, hyper-sphere), both with (bottom row) and without (top row) affine covariance adaptation. The plots show the radius of a hypercube/hypersphere such that approximately $\{0.35\%, 0.55\ \%, 0.75\%, 0.95\%\}$ of the points in this body are feasible, as indicated by the target hitting probabilities at the top of each plot. The larger this radius, the more robust the design center.

Unorthodox system

In the unorthodox TCS, HK has three phosphorylation sites: H1, D1, and H2. This leads to eight different phospho-species of HK, $HK_{1...8}$, with the following phosphorylation states (1=phosphorylated, 0=unphosphorylated):

	H1	D1	H2
HK_1	1	1	1
HK_2	0	1	1
HK_3	1	0	1
HK_4	1	1	0
HK_5	0	0	1
HK_6	0	1	0
HK_7	1	0	0
HK_8	0	0	0

Again it is assumed that the total concentrations of HK and RR sum up to 1:

$$\sum_{i=1}^{8} HK_i = 1$$
$$RR + RR_p = 1$$

This leads to the following model equations for the time dynamics of the concentrations $[\cdot]$ of all involved chemical species:

$$\frac{d[HK_1]}{dt} = k_4[HK_4](1-[RR_p]) + k_6[HK_3] - k_7[HK_1][RR_p] + k_8[HK_2] - k_1[HK_1][S]$$
$$\frac{d[HK_2]}{dt} = k_4[HK_6](1-[RR_p]) + k_6[HK_5] - k_7[HK_2][RR_p] - k_8[HK_2] + k_1[HK_1][S] - k_2[HK_2]$$
$$\frac{d[HK_3]}{dt} = -k_3[HK_3] + k_4[HK_7](1-[RR_p]) + k_5[HK_4] - k_6[HK_3] - k_7[HK_3][RR_p] + k_8[HK_5] - k_1[HK_3][S] + k_2[HK_2]$$
$$\frac{d[HK_4]}{dt} = k_3[HK_3] - k_4[HK_4][1-RR_p] - k_5[HK_4] + k_6[HK_7] + k_7[HK_1][RR_p] + k_8[HK_6] - k_1[HK_4][S]$$
$$\frac{d[HK_5]}{dt} = -k_3[HK_5] + k_4(1-\sum_{i=1}^{7}[HK_i])(1-[RR_p]) + k_5[HK_6] - k_6[HK_5] - k_7[HK_5][RR_p] - k_8[HK_5] + k_1[HK_3][S]$$
$$\frac{d[HK_6]}{dt} = k_3[HK_5] - k_2[HK_6] - k_4[HK_6](1-[RR_p]) - k_5[HK_6] + k_6(1-\sum_{i=1}^{7}[HK_i]) + k_7[HK_2][RR_p] - k_8[HK_6] + k_1[HK_4][S]$$
$$\frac{d[HK_7]}{dt} = k_2[HK_6] - k_4[HK_7](1-[RR_p]) - k_6[HK_7] + k_7[HK_3][RR_p] + k_8(1-\sum_{i=1}^{7}[HK_i]) - k_1[HK_7][S]$$
$$\frac{d[RR_p]}{dt} = k_4(1-[RR_p])(1-[HK_1]-[HK_2]-[HK_3]-[HK_5]) - k_7[RR_p]([HK_1]+[HK_2]+[HK_3]+[HK_5]) - k_9[RR_p].$$

Note that in the original paper[13] the equation for $\frac{d[HK_5]}{dt}$ has a typo. The first term should be $-k_3[HK_5]$, as shown above, and not $-k_3[HK_3]$.

Membership oracles

The membership oracle numerically solves the dynamic equations in order to decide whether a given vector $\mathbf{x} \in \mathbb{R}^n$ ($n = 5$ for the orthodox system, $n = 9$ for the unorthodox system) of kinetic variables leads to a reaction network that fulfills the specifications, or not. The specifications are given in terms of the input/output behavior of the network. The output is the concentration of RR_p in response to a specific input signal S over time points $T = \{t_k\}_{1\leq k\leq N}$ where $t_1 = 0$, $t_N = 10$, and $t_{k+1} = t_k + 0.001$. We consider four different cases, corresponding to different network design goals and hence different membership oracles, as described below.

fast response

The "fast response" case aims to design a network that rapidly follows a sudden change of the input. The following input signal is considered, which has two sudden changes (cf. Fig. 8(a) in the main text):

$$[S] = \begin{cases} 1, & \text{if } t_k \in [2,4] \\ 0, & \text{else.} \end{cases}$$

Desired output: The network fulfills the specifications if the maximum response of the output is reached within 0.1 time units after the pulse starts, and the minimum no more than 0.1 time units after the pulse ends. The specifications hence are (cf. Fig. 8(b) in the main text):

$$t_{\max} = \arg\max_k [RR_p](t_k)$$
$$t_{\min} = \arg\min_k [RR_p](t_k > t_{\max})$$

where $\mathbf{x}$ is feasible iff $0 \leq t_{\max} - 2 \leq 0.1$ and $0 \leq t_{\min} - 4 \leq 0.1$.

steady output

The "steady output" case aims to design a network that produces a stationary output upon a constant input signal of the same magnitude. We consider the constant input

$$[S] = 1.$$

Desired output: The network fulfills the specifications if the output is steady at the same level at most 2 time units after the input appeared. The specifications are:

$$\varepsilon = \frac{N_B}{N_2} \sum_{\substack{k \\ t_k \geq 2}} \left([RR_p](t_k) - 1\right)^2,$$

where N_2 is the number of elements in $T = \{t_k\}, t_k \geq 2$, and $N_B = 160$ is the number of elements used by Barnes[13]. The vector $\mathbf{x}$ is feasible iff $\varepsilon \leq 0.01$.

noise rejection

The "noise rejection" case aims to design a network that removes high frequencies from the input signal. We consider the input signal (cf. Fig. 8(c) in the main text):

$$[S] = 0.5 + 0.4 \cdot \sin(8\pi t),$$

consisting of a constant (DC) part and an oscillatory (AC) signal. Desired output: The network fulfills the specifications if the output signal rejects the oscillatory (AC) part after at most 2 time units. The specifications hence are:

$$\varepsilon = \frac{N_B}{N_2} \sum_{\substack{k \\ t_k \geq 2}} \left([RR_p](t_k) - 0.5\right)^2$$

with the same definitions as above. The vector $\mathbf{x}$ is feasible iff $\varepsilon \leq 0.3$.

signal reproduction

The "signal reproduction" case aims to design a network where the output reproduces the input within a certain tolerance. We again consider as input the square pulse (cf. Fig. 8(d) in the main text):

$$[S] = \begin{cases} 1, & \text{if } t_k \in [2,4] \\ 0, & \text{else.} \end{cases}$$

Desired output: The network fulfills the specifications if the output signal rises above 0.8 within 1 time unit after the pulse starts, and drops to below 0.2 within 1 time unit after the pulse ends. The specifications hence are:

$$t_{\max} = \arg\max_k [RR_p](t_k)$$
$$t_{\min} = \arg\min_k [RR_p](t_k > t_{\max})$$

where $\mathbf{x}$ is feasible iff $0 \leq t_{\max} - 2 \leq 1$ and $0 \leq t_{\min} - 4 \leq 1$ and $(1 - \max([RR_p])) < 0.2$ and $\min([RR_p](t_k > t_{\max})) < 0.2$.

Supplementary Figure 6 shows the evolution of the estimated normalized volumes of the feasible regions versus the number of function evaluations for all cases of both TCS models. The normalized volume is a measure for the robustness of the system, quantifying the subspace of rate constants for which the system fulfills the specifications. The hitting probability is dynamically reduced according to the schedule shown on top of each plot. The dashed black line shows the baseline result obtained by exhaustive brute-force sampling the orthodox system $7.5 \cdot 10^6$ times and the unorthodox system $1.5 \cdot 10^7$ times. L_p-Adaptation

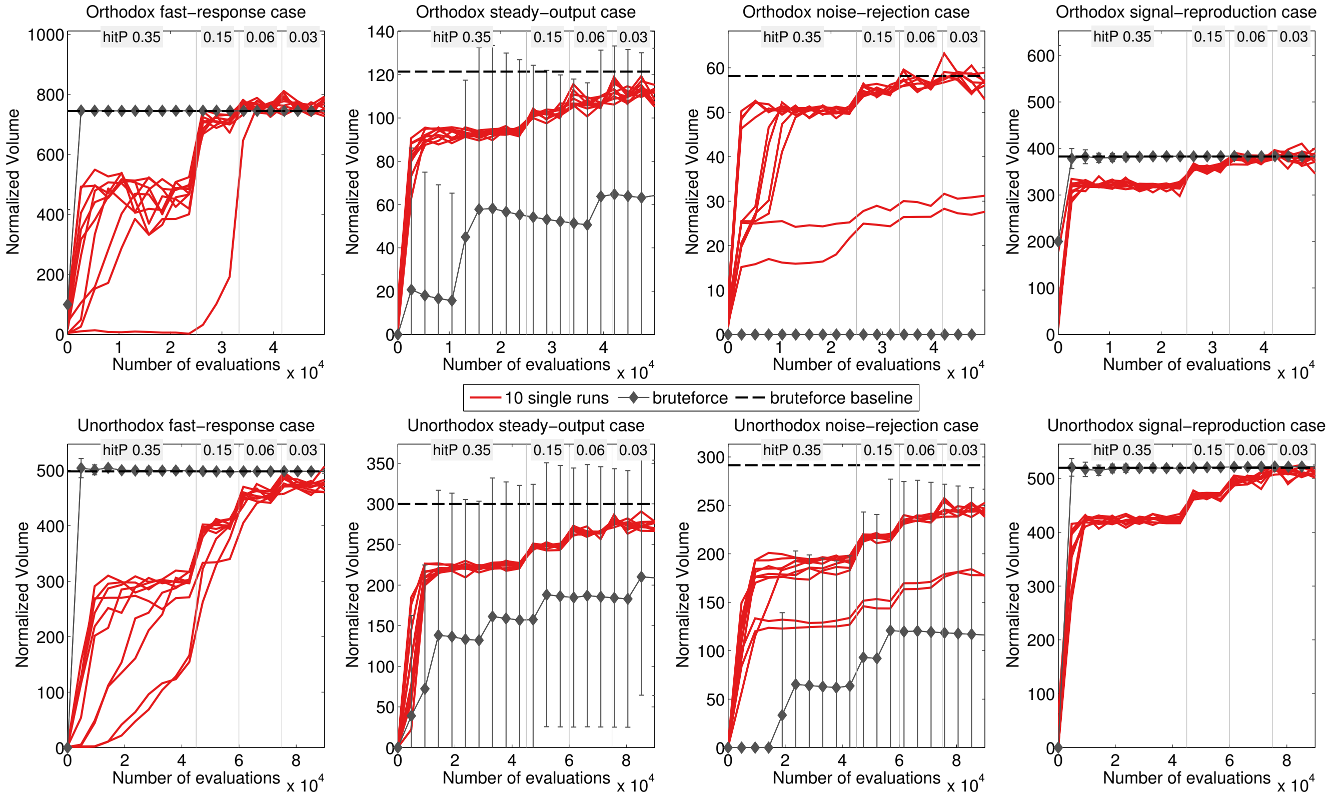

Supplementary Figure 6. Normalized volume ($\sqrt[n]{V}$) estimation of the feasible regions of all four cases for both TCS models. The 10 independent runs of L_p-Adaptation using an L_2-ball proposal are shown as individual red lines in each case. The ground-truth baseline obtained by exhaustive brute-force sampling is shown as a dashed black line. Brute-force sampling using the same number of function evaluations as L_p-Adaptation is shown with gray diamonds and error bars (standard deviation over 10 brute-force runs). The schedule of reducing the target hitting probability is shown at the top of each plot.

converges toward the baseline in all cases. In the noise-rejection cases, however, the different runs cover different parts of the non-convex feasible region and some of them do not converge to the baseline. Cases where brute-force sampling reaches the baseline faster are indicative of a feasible region that fills almost the entire space, as also confirmed by the larger normalized volumes in these cases (fast-response and signal-reproduction cases). The volume of the entire parameter space is 1000 in this example. When the feasible region is significantly smaller than the whole space, L_p-Adaptation performs better and more reliably than brute-force sampling (steady-output and noise-rejection cases). The orthodox noise-rejection case has a particularly small feasible region. In this case, none of the 10 brute-force sampling runs finds any feasible solution, whereas L_p-Adaptation reaches the baseline in 8 out of 10 runs.

Marginal distributions

We provide additional figures of the marginal and pairwise distributions of parameters for the TCS. Supplementary Figure 7 shows the joint pairwise distributions and the marginal densities of the feasible points of the unorthodox system for the noise-rejection case. Comparing the distributions with those shown in Barnes' appendix, Figure 5 of Ref.[13], we see that our method explores the parameter space more comprehensively.

Supplementary Figure 8 compares the marginal distributions obtained by approximate Bayesian computation based on sequential Monte Carlo (ABC)[13], L_p-Adaptation, and brute-force sampling. Using brute-force sampling, 228 feasible points were found by sampling $1.5 \cdot 10^7$ points. L_p-Adaptation finds 159,140 feasible points by sampling $9 \cdot 10^5$ points in 10 runs, each with different a starting point. This indicates that L_p-Adaptation provides an efficient way of exploring feasible regions that are small compared to the total space. Using only 6% of the function evaluations of brute-force sampling, the number of feasible points found by L_p-Adaptation is almost 700-times higher than that of brute-force sampling, amounting to five orders of magnitude better sampling efficiency.

Supplementary Figure 9 shows the joint pairwise distributions and the marginal densities of the feasible points of the orthodox system for the signal-reproduction case. This is the same case also shown by Barnes *et al.*[13]. Supplementary Figure 10

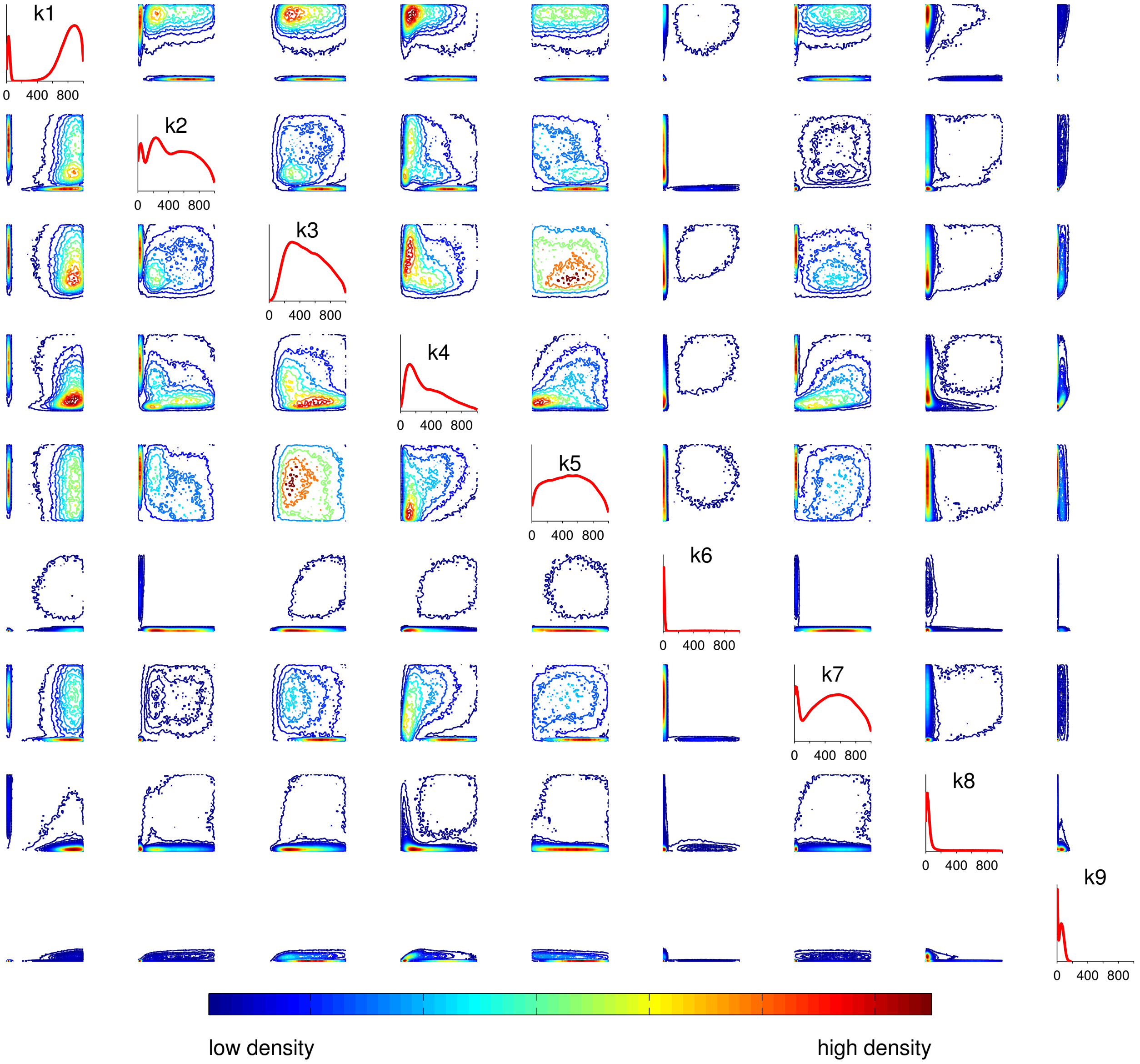

Supplementary Figure 7. Marginal densities (diagonal) and joint pairwise distributions of feasible points for the unorthodox TCS in the noise-rejection case.

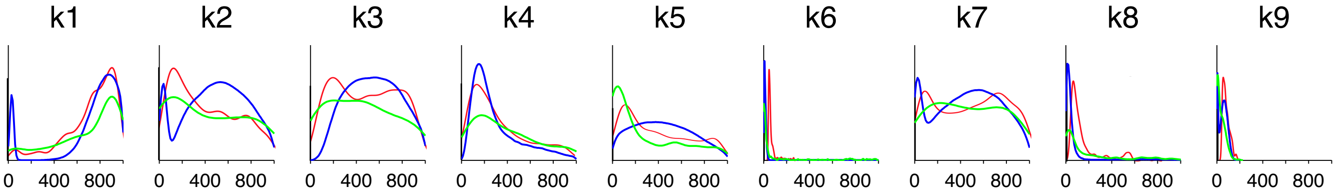

Supplementary Figure 8. Comparison of the marginal densities for each of the nine design parameters of the unorthodox TCS in the noise-rejection case. Red: marginals obtained by approximate Bayesian computation based on sequential Monte Carlo (ABC)[13]; blue: marginals obtained by L_p-Adaptation; green: marginals from points obtained by uniformly sampling the entire parameter space (brute-force sampling).

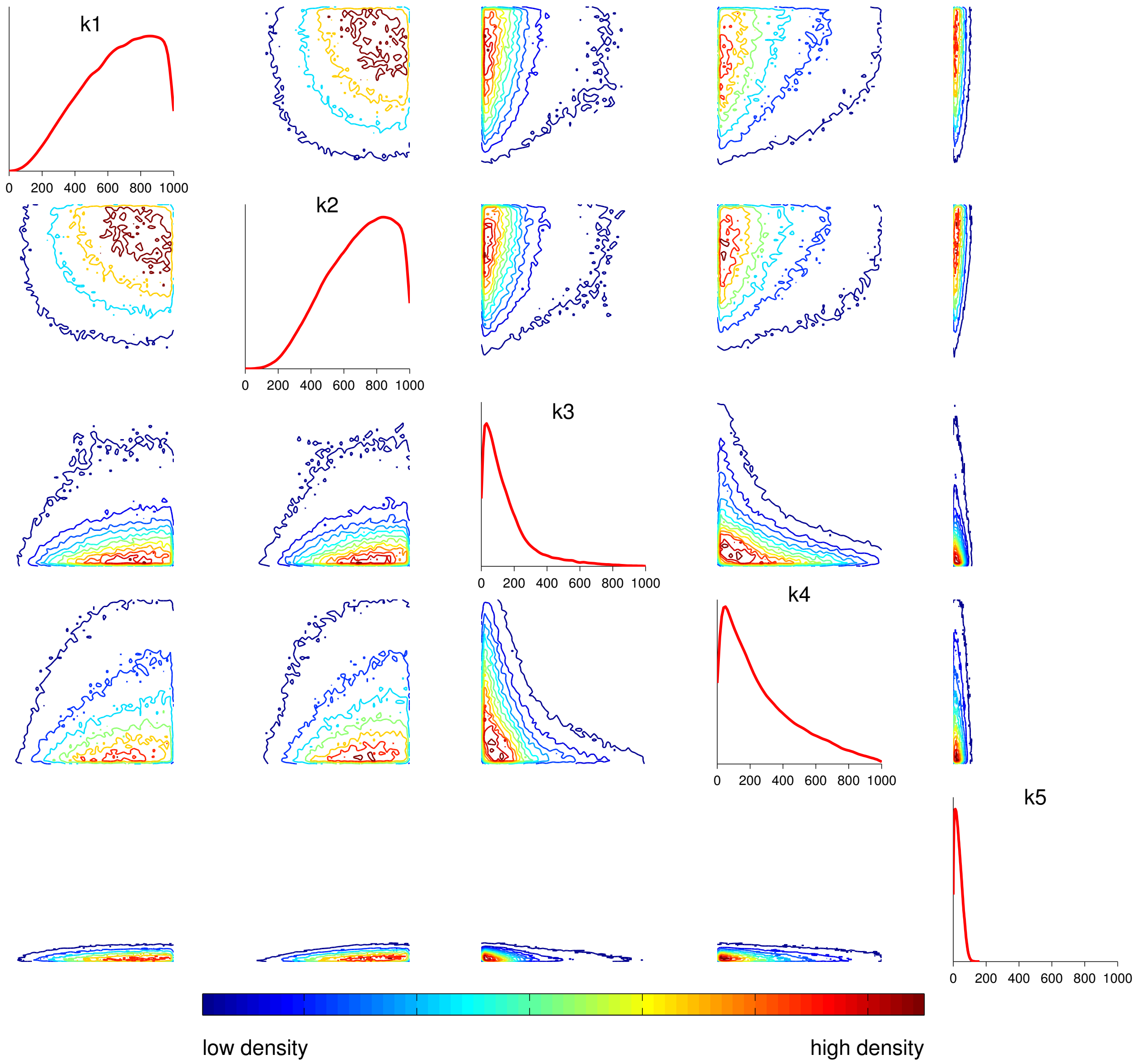

Supplementary Figure 9. Marginal densities (diagonal) and joint pairwise distributions of feasible points of the orthodox TCS in the signal-reproduction case.

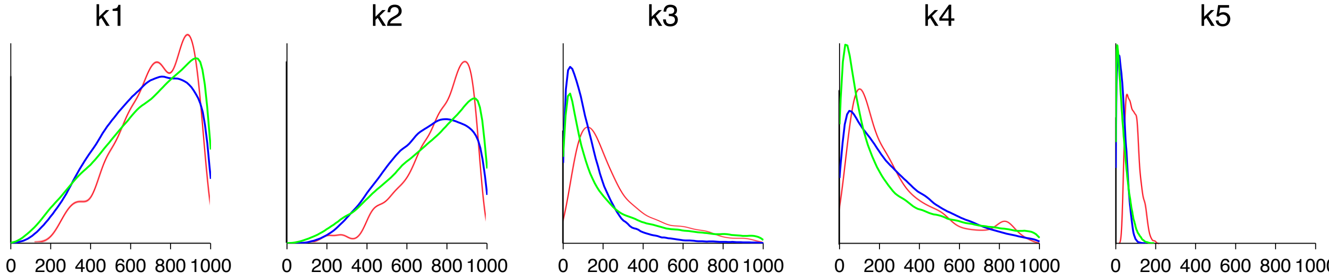

Supplementary Figure 10. Comparison of the marginal densities for each of the five design parameters of the orthodox TCS in the signal-reproduction case. Red: marginals obtained by approximate Bayesian computation based on sequential Monte Carlo (ABC)[13]; blue: marginals obtained by L_p-Adaptation; green: marginals from points obtained by uniformly sampling the entire parameter space (brute-force sampling).

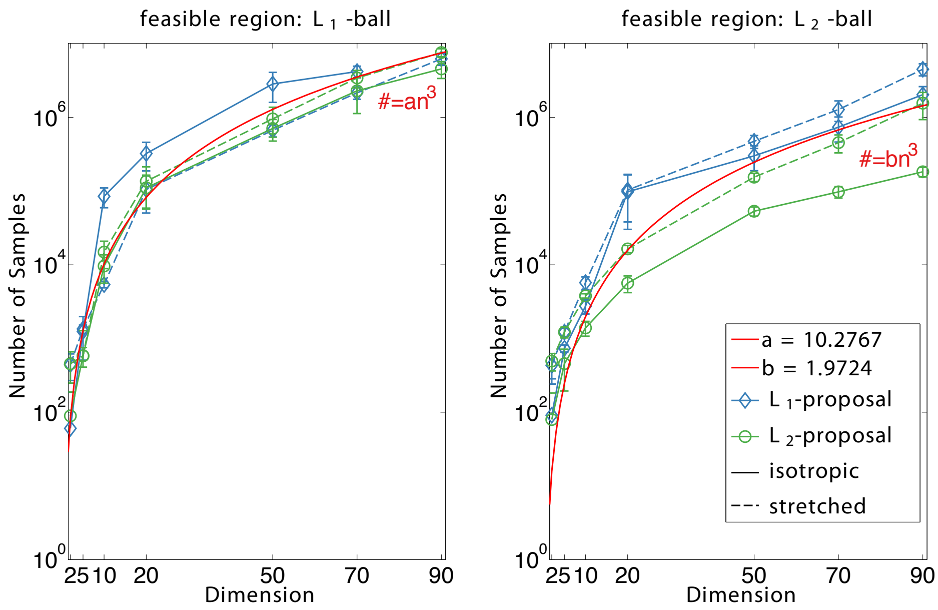

Supplementary Figure 11. Number of samples (oracle evaluations) required in increasing space dimension n to reach a relative volume approximation error of less than 0.1 for isotropic (solid lines) and stretched (dashed lines) L_1 and L_2-balls, respectively. The red lines show the best least-squares fits of a cubic scaling.

again compares the marginal densities obtained by the three different methods. The conclusions are commensurate with those from the orthodox system.

Computational Complexity

Supplementary Figure 11 shows the number of oracle evaluations required to reach a relative volume-approximation error < 0.1 vs. the dimension n of the space. The relative error is defined as $|\text{trueVol - estimatedVol}|/\text{trueVol}$. Results are shown for both stretched (dashed lines) and isotropic (solid lines) L_1 and L_2-balls as feasible regions. The red lines are the best least-squares fits of a cubic scaling, i.e., the curve expected if the number of samples # required scales with n^3. The respective pre-factors are given in the inset legend, suggesting that the L_1 case is harder than the L_2 case.

Supplementary References

1. Kjellström, G. & Taxen, L. Stochastic optimization in system design. *IEEE Trans. Circ. and Syst.* **28**, 702–715 (1981).
2. Müller, C. L. & Sbalzarini, I. F. Gaussian Adaptation for robust design centering. In Poloni, C., Quagliarella, D., Périaux, J., Gauger, N. & Giannakoglou, K. (eds.) *Evolutionary and deterministic methods for design, optimization and control, Proc. EuroGen*, 736–742 (CIRA, ECCOMAS, ERCOFTAC, Capua, Italy, 2011).
3. Hansen, N. & Ostermeier, A. Adapting Arbitrary Normal Mutation Distributions in Evolution Strategies: The Covariance Matrix Adaptation. In *Proceedings of the 1996 IEEE Conference on Evolutionary Computation (ICEC '96)*, 312–317 (1996).
4. Hansen, N. Adaptive encoding for optimization (2008).
5. Seifi, A., Ponnambalam, K. & Vlach, J. A unified approach to statistical design centering of integrated circuits with correlated parameters. *Circuits and Systems I: Fundamental Theory and Applications, IEEE Transactions on* **46**, 190–196 (1999).
6. Müller, C. L. & Sbalzarini, I. F. Gaussian Adaptation as a unifying framework for continuous black-box optimization and adaptive Monte Carlo sampling. In *Proc. IEEE Congress on Evolutionary Computation (CEC)*, 2594–2601 (Barcelona, Spain, 2010).
7. Calafiore, G., Dabbene, F. & Tempo, R. Uniform sample generation in l p balls for probabilistic robustness analysis. In *Decision and Control, 1998. Proceedings of the 37th IEEE Conference on*, vol. 3, 3335–3340 (IEEE, 1998).
8. Gruber, P. M. John and loewner ellipsoids. *Discrete & Computational Geometry* **46**, 776–788 (2011).

9. Khachiyan, L. G. Rounding of polytopes in the real number model of computation. *Mathematics of Operations Research* **21**, 307–320 (1996).

10. Lasserre, J. Unit balls of constant volume: which one has optimal representation? *arXiv preprint arXiv:1408.1324* (2014).

11. Henrion, D., Lasserre, J. B. & Savorgnan, C. Approximate volume and integration for basic semialgebraic sets. *SIAM review* **51**, 722–743 (2009).

12. Storn, R. System design by constraint adaptation and differential evolution. *IEEE Transactions on Evolutionary Computation* **3**, 22–34 (1999).

13. Barnes, C. P., Silk, D., Sheng, X. & Stumpf, M. P. Bayesian design of synthetic biological systems. *Proceedings of the National Academy of Sciences* **108**, 15190–15195 (2011).

14. Kim, J.-R. & Cho, K.-H. The multi-step phosphorelay mechanism of unorthodox two-component systems in e. coli realizes ultrasensitivity to stimuli while maintaining robustness to noises. *Computational biology and chemistry* **30**, 438–444 (2006).

15. Schmidt, H. & Jirstrand, M. Systems biology toolbox for matlab: a computational platform for research in systems biology. *Bioinformatics* **22**, 514–515 (2006).
